# Heavily Oiled Salt Marsh following the *Deepwater Horizon* Oil Spill, Ecological Comparisons of Shoreline Cleanup Treatments and Recovery

**DOI:** 10.1371/journal.pone.0132324

**Published:** 2015-07-22

**Authors:** Scott Zengel, Brittany M. Bernik, Nicolle Rutherford, Zachary Nixon, Jacqueline Michel

**Affiliations:** 1 Emergency Response Division, Office of Response and Restoration, National Ocean Service, National Oceanic and Atmospheric Administration (NOAA), Seattle, Washington, United States of America; 2 Research Planning, Inc. (RPI), Tallahassee, Florida, United States of America; 3 Department of Ecology and Evolutionary Biology, Tulane University, New Orleans, Louisiana, United States of America; 4 Research Planning, Inc. (RPI), Columbia, South Carolina, United States of America; University of California, Merced, UNITED STATES

## Abstract

The *Deepwater Horizon* oil spill affected hundreds of kilometers of coastal wetland shorelines, including salt marshes with persistent heavy oiling that required intensive shoreline “cleanup” treatment. Oiled marsh treatment involves a delicate balance among: removing oil, speeding the degradation of remaining oil, protecting wildlife, fostering habitat recovery, and not causing further ecological damage with treatment. To examine the effectiveness and ecological effects of treatment during the emergency response, oiling characteristics and ecological parameters were compared over two years among heavily oiled test plots subject to: manual treatment, mechanical treatment, natural recovery (no treatment, oiled control), as well as adjacent reference conditions. An additional experiment compared areas with and without vegetation planting following treatment. Negative effects of persistent heavy oiling on marsh vegetation, intertidal invertebrates, and shoreline erosion were observed. In areas without treatment, oiling conditions and negative effects for most marsh parameters did not considerably improve over two years. Both manual and mechanical treatment were effective at improving oiling conditions and vegetation characteristics, beginning the recovery process, though recovery was not complete by two years. Mechanical treatment had additional negative effects of mixing oil into the marsh soils and further accelerating erosion. Manual treatment appeared to strike the right balance between improving oiling and habitat conditions while not causing additional detrimental effects. However, even with these improvements, marsh periwinkle snails showed minimal signs of recovery through two years, suggesting that some ecosystem components may lag vegetation recovery. Planting following treatment quickened vegetation recovery and reduced shoreline erosion. Faced with comparable marsh oiling in the future, we would recommend manual treatment followed by planting. We caution against the use of intensive treatment methods with lesser marsh oiling. Oiled controls (no treatment “set-asides”) are essential for judging marsh treatment effectiveness and ecological effects; we recommend their use when applying intensive treatment methods.

## Introduction

**Fig 1:**
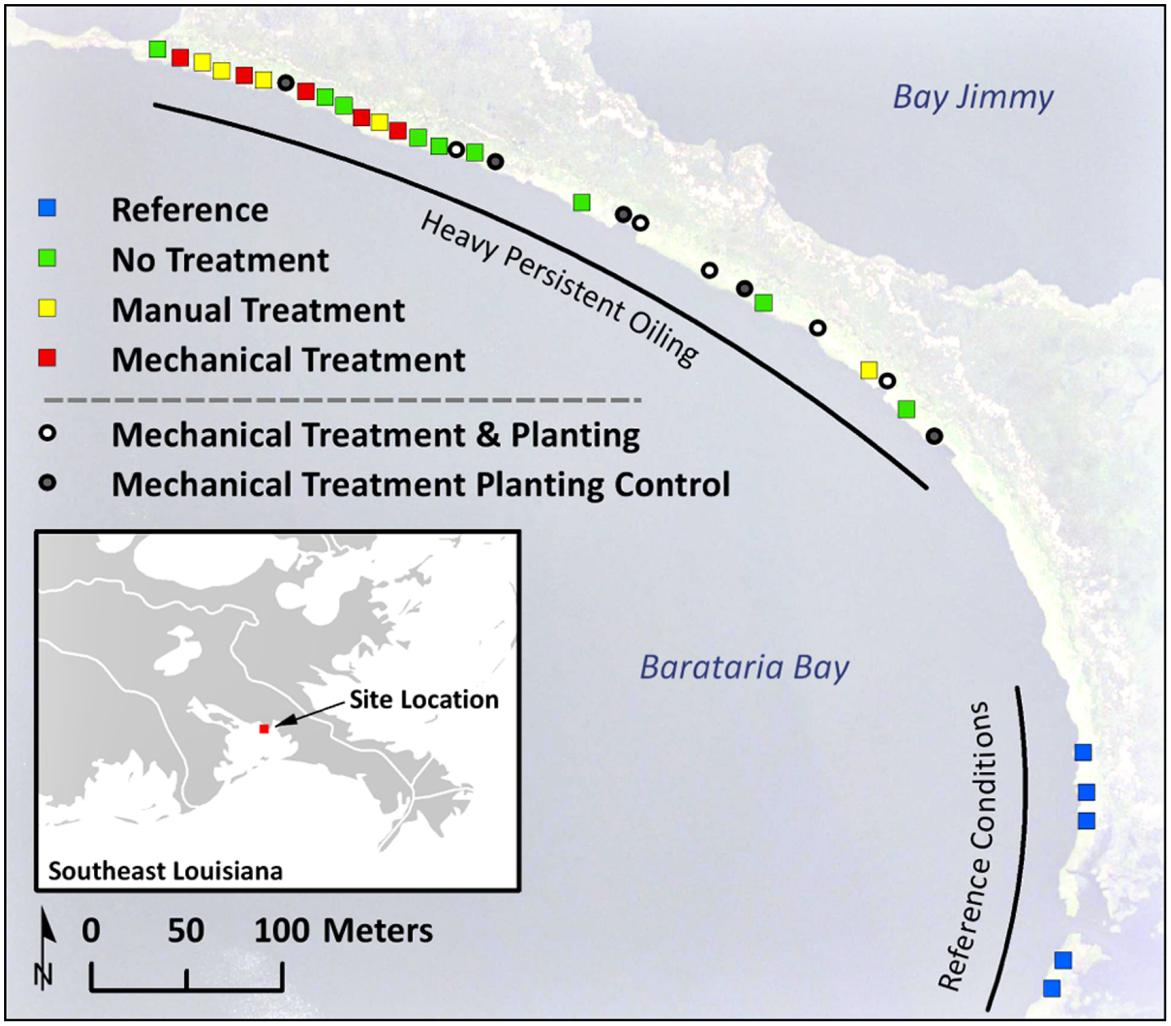
Study area map, including plot locations by oiling/treatment class.

The *Deepwater Horizon* oil spill resulted in the oiling of 796 kilometers (km) of coastal marsh shorelines according to Shoreline Cleanup Assessment Technique (SCAT) surveys during the emergency response [1]. Of this total, 135 km of shoreline were described as heavy marsh oiling, based on a combination of oiling width across the shore, oiling percent cover, and oil thickness [1]. Persistent heavy oiling was most widespread in salt marshes in northern Barataria Bay, Louisiana, in marshes dominated primarily by *Spartina alterniflora*, and to a lesser extent by *Juncus roemerianus* [1–4]. Due to the degree and nature of oiling in this area, typical low-intensity “cleanup” treatments, including use of sorbents and low-pressure water flushing, were not effective for the most heavily oiled marshes [2], presenting continuing oil remobilization and exposure risks for adjacent habitats and wildlife (see [5] for an overview of oiled marsh treatment methods). In addition, due to the degree of heavy oiling, and experience from prior spills with similar oiling, there was concern that the long-term recovery of these marshes could be at risk without treatment, due primarily to the presence of thick emulsified oil layers, which can be very slow to degrade in coastal wetland environments [5–8]. At the same time, there was the competing concern that aggressive oil removal, such as manual or mechanical cutting, raking, or scraping, could cause further marsh damage, delaying or limiting marsh recovery, as has often been observed following oil spills [5,9–14]. Due to these multiple concerns, we conducted field experiments to evaluate treatment options prior to wider-scale treatment application [2]. The treatment tests were conducted on heavily oiled marsh shoreline centrally located in the affected area, near Bay Jimmy in northern Barataria Bay (Fig 1; 29.443899° N, 89.887604° W). The tests and subsequent monitoring led to the development and adaptive improvements of operational-scale Shoreline Treatment Recommendations (STRs) implemented under the emergency spill response across 11 km of shoreline in northern Barataria Bay [2] and in smaller areas with similar oiling in Terrebonne-Timbalier Bay and Biloxi Marsh (Chandeleur Sound), Louisiana [1].

This paper examined the effectiveness and ecological effects of different oiled marsh “cleanup” treatment methods used in the treatment test area. Oiling characteristics and several ecological parameters were compared among manual treatment, mechanical treatment, natural recovery, and adjacent reference conditions over two years following initial oil impact. Manual treatment was conducted by small crews using hand tools to remove oil and oiled debris to the degree possible and expose residual oiling to natural degradation processes. Mechanical treatment used mechanized tools aimed towards the same goals as manual treatment with anticipated increases in efficiency and fewer personnel in the marsh. Natural recovery involved “no treatment”, serving as both an emergency response option frequently used for oiled marshes and as an oiled control, to allow more meaningful evaluation of active treatment methods. Collectively, the manual, mechanical, and no treatment sampling areas comprised the “heavily oiled plots” as compared to the adjacent reference plots which had lighter to no oiling, intact vegetation structure, and no treatment. The reference plots represented target conditions for defining recovery in the heavily oiled plots.

Ecological comparisons examined the marsh vegetation, which defines the salt marsh and serves as the foundation species for this habitat. Salt marsh vegetation can be variably affected by oil spills, but, impacts can be more severe, and recovery times longer, when the majority of the plant stems and leaves are coated with oil and the marsh substrate is also heavily oiled [5,15], as was the case during our study. See Michel and Rutherford (2014) [5] and Mendelssohn et al. (2012) [15] for recent reviews of marsh vegetation impacts and recovery following oil spills. Ecological comparisons also included two common intertidal macroinvertebrates, marsh periwinkles (*Littoraria irrorata*) and fiddler crabs (*Uca* spp.). These taxa are also important to marsh structure and function, and we were interested in looking at these species so that our definitions of impact and recovery were not limited to just the vegetation, as is commonly the case during emergency response. Marsh periwinkles can be affected by oil spills, with impacts including increased mortality, reduced density, reduced recruitment, and altered size distributions [16–18]. Oil spill impacts to fiddler crabs can include increased mortality, reduced burrow density, impaired locomotion and behavior, abnormal burrow construction, changes in sex ratio, and reduced juvenile settlement [19–22]. We also examined marsh shoreline retreat (erosion) to determine whether oiling or treatment influenced shoreline erosion and marsh loss, which are major concerns in coastal Louisiana [23–24]. Finally, in an additional experiment, we compared areas with and without vegetation planting following mechanical treatment, examining the influence of planting on vegetation recovery and shoreline retreat.

Our study provided a field experiment during a spill of national significance, examining existing spill response guidance from prior case histories. This guidance was best summarized by Baker (1999) [13] for salt marshes with thick oil deposits (see also [5,11]):

“Because neither natural cleanup nor aggressive treatment provides the best environmental benefit, it seems that the greatest benefit would result from a moderate level of clean-up—sufficient to remove most of the bulk oil, but gentle enough to leave the surface of the shore intact and to avoid churning oil into underlying sediments. This can be achieved by using small crews and avoiding the use of heavy machinery as far as possible. The appearance of the shore after such treatment is likely to be somewhat oily and therefore not optimal from an aesthetic viewpoint, but there are numerous examples of biological recovery taking place in the presence of weathered oil remnants. If plants were smothered to death before removal of the bulk oil, replanting schemes can be helpful.”

Our work also supplements earlier and ongoing studies of *Deepwater Horizon* oil spill impacts and recovery in oiled marshes [25–33], adding a focused consideration of shoreline treatment effectiveness and effects. In addition, only a limited number of field studies and case histories have evaluated vegetation planting in oiled marshes following spills [10,16,34–35]. Our study further evaluated whether planting vegetation would be an effective strategy to enhance vegetation recovery and marsh stability following oil impacts and intensive shoreline treatments.

Our overall study objective was to examine and compare marsh recovery under heavy oiling conditions with and without different shoreline treatments over the first two years following oil impact. The treatment tests and subsequent monitoring informed the emergency spill response [2], serving as another example of “science in support of the *Deepwater Horizon* response” [36]. Our study was also closely aligned with the types of emergency response studies called for by Peterson et al. (2012) [37], which are often lacking during spills. Sharing this information will be useful for other scientific endeavors associated with the *Deepwater Horizon* spill, as well as for oil spill planning, emergency response, damage assessment, and restoration for future spills.

## Methods

### Treatments and Plot Set-up

Manual treatment involved raking, cutting, and removal of oiled wrack, oiled vegetation mats, and underlying oil on the substrate by small crews using hand tools (see [2] for photos of manual treatment and additional details). Hand crews used walking boards to minimize foot traffic on the marsh surface. The manual treatments formed the basis of the STRs. Mechanical treatment involved mechanized grappling to remove oiled wrack and mechanized raking, cutting, and scraping to remove or reduce oiled vegetation mats and oil on the substrate, followed by additional manual treatment and loose natural sorbent application. The mechanical treatments were applied using long-reach hydraulic arms mounted on shallow-draft barges and large airboats stationed just seaward of the marsh shoreline (see [2] for photos of mechanical treatment and additional details). The natural recovery (no treatment, oiled control) and reference plots were not treated.

Sampling plots were ~50 square meters (m^2^) each, spanning the width of heavy oiling across shore. The manual treatment plots (5 replicates) and no treatment plots (9 replicates) were randomly established within the continuous heavy oiling band during October to December 2010. The manual treatments were applied in December 2010. The no treatment plots were left untreated for the duration of the study. The no treatment plots represented the only comparably oiled sites in the study area where shoreline treatment was not applied. The mechanical treatment plots (5 replicates) were randomly established in areas that received operational-scale mechanical treatments in May-June 2011 under the emergency response. The reference plots (5 replicates) were randomly located along the nearest contiguous and comparable section of shoreline in the study area (with minor to no oiling during the treatment tests). Although it would have been desirable to have reference plots randomly interspersed among the heavily oiled and variously treated plots, this was impossible due to the distribution of heavy oiling. It would also have been desirable to apply the manual and mechanical shoreline treatments at the same time, however, this was impossible as this work was part of an on-going emergency response, and the mechanical treatment methods were developed and applied at a later time (described above) by the emergency response organization. The time difference was taken into account when interpreting the data.

As part of a separate experiment, planting of *S*. *alterniflora* was conducted following the operational mechanical treatments. For the planting experiment, ~20 m^2^ plots were randomly established within the mechanically treated areas during July-August 2011 [38] (following mechanical treatment in May-June 2011, as described above). Both planted and unplanted (control) plots were randomly assigned (5 replicates each). For the planting experiment, both the planted and control plots were set back from the seaward marsh edge by 4.5 m at the time they were installed, so that the plantings would have time to establish prior to encountering shoreline retreat. Individual bare root *S*. *alterniflora* stems were planted by hand, using a transplanted *S*. *alterniflora* variety propagated from wild stocks native to Bay Jimmy. Fifty-five stems were planted approximately 45 centimeters (cm) apart along five rows: four rows running shore normal spaced on 90 cm centers, and the fifth row running parallel to shore along the interior or “landward” edge of the plots (resulting in a planting density of 2–3 stems m^-2^). Planting was completed in late summer and early fall 2011. No fertilizer was used during planting. Naturally recruiting aboveground vegetation other than *S*. *alterniflora* was trimmed by hand during planting and subsequently in planted areas to limit competition with planted material. The control areas were trimmed as well for consistency (though minimal trimming was needed overall).

### Oiling Conditions

Multiple pre-treatment oiling assessments were conducted across the study area during June-December 2010. The earliest pre-treatment oiling assessments were SCAT surveys spanning the study area and surrounding region. Later pre-treatment assessments were conducted at plot level. Where assessments were conducted by multiple personnel near the onset of the study, observations from the longest-term participant were given priority, for consistency and continuity with the post-treatment assessments. Mechanical treatment plots were not yet established during the pre-treatment assessments. However, each future mechanical plot fell between two existing plots. After reviewing the oiling assessment data, as well as overlapping plot photographs depicting oiling conditions, we determined that it was appropriate to estimate pre-treatment oiling values in the mechanical plots by averaging the two nearest pre-existing plots on either side. Post-treatment oiling assessments were conducted in September 2011 and 2012 for all the plots. Surface and subsurface oiling descriptions were based on SCAT methods applied individually to each plot, including estimates of oiling width across shore, oil percent cover (%), oil thickness, and oil character across the entire plot [39]. Oiling assessments included at least three shovel test pits per plot to measure oil thickness, assist in examining oil on the marsh substrate beneath the oiled vegetation mats, and to describe subsurface oiling.

Subsurface soil sampling was conducted during July-August 2011 and September 2012. Soil samples were collected using 15 cm diameter cores taken to 10 cm depth, specifically excluding oiled vegetation debris and oil on the marsh surface, which was carefully scraped aside where necessary prior to sampling. Therefore, our oil chemistry results represent oil concentrations in the subsurface soils (not oil on the marsh surface). One soil core was collected from the approximate center of each plot. Total polycyclic aromatic hydrocarbons (tPAH) in marsh soils were determined using GC/MS-SIM (gas chromatography/mass spectrometry in selective ion monitoring mode) based on EPA Method 8270D [40]. TPAH included the sum of 43–45 PAHs, including alkylated homologues, presented as mg/kg.

### Ecological Parameters

Ecological sampling was conducted primarily in September 2011 and 2012 (vegetation sampling in the planting experiment was conducted in July 2012). Vegetation percent cover (%) was estimated visually across each plot in total and for each plant species observed. Marsh periwinkles and fiddler crab burrows were counted in three 0.25 m^2^ quadrats along the approximate centerline of each plot, with one quadrat each located near the seaward marsh edge (~1.5 m from edge), near the center of the plot, and near the landward extent of the plot. Data were converted to m^-2^ basis for analysis. In 2012, periwinkle counts included careful searches for the smallest juvenile snails hidden between the vegetation leaf sheaths and stems or in rolled leaves. All marsh periwinkles recorded in the quadrats in 2012 were measured using digital calipers to determine total shell length to the nearest 1 millimeter (mm). Shell length data were used to compare size frequency histograms among treatments, including periwinkle life-history stages based on shell length; defined as juveniles (<6 mm), sub-adults (6–13 mm), and adults (>13 mm) (after [41–42]). Fiddler crab species composition was determined by collecting fiddler crabs on the marsh surface and from burrow entrances within each plot to make species identifications. A maximum of 10 fiddler crabs were captured and identified to species for each plot.

Shoreline retreat measurements were made using the plot stakes established during plot set-up. Stakes were placed at the seaward edge of the marsh platform (defined by an erosional scarp) and at a back stake along both sides of each plot. Stake positions were recorded using post-processed differentially corrected Global Positioning System (GPS) locations (±10 cm), and these positions were used to create digital cross-shore transects via a Geographic Information System (GIS) (two transects per plot). Back stake locations were confirmed during each sampling period by GPS and changes in the location of the seaward marsh edge (erosional scarp) were re-staked and recorded via GPS along the digital transects. During initial set-up and subsequent annual sampling (November 2011 and September 2012), the distance between the back stakes and the stakes at the seaward marsh edge were directly measured using a tape measure. When stakes were missing, distance measurements made in the field to previously recorded locations using GPS were used in lieu of tape measurements. Methods were the same for the planting plots; however, as these plots were set back from the marsh edge, transect measurements extended beyond the plot stakes to the seaward marsh edge. The differences in cross-shore distances between the back stake and seaward marsh edge in each subsequent year were averaged across the two transects per plot. The annual shoreline retreat rate was calculated by dividing the shoreline retreat distance by the number of days between measurements then multiplying by 365 days, expressed as meters per year (m yr^-1^).

### Data Analysis and Availability

Repeated measures ANOVA was used for all data collected in both 2011 and 2012. For data collected in only a single year, ANOVA with Tukey HSD comparisons, or t-tests, were used depending on the number of classes being compared. We were primarily focused on differences among oiling/treatment classes over the duration of the study; however, we also report p-values for the effects of year and the interaction of oiling/treatment class and year. We defined p ≤ 0.05 as indicating statistical significance. We report all p-values to two decimal places. In cases where p = 0.00 due to rounding, we report p < 0.01. Because replication was modest and we wanted to be conservative in terms of not falsely dismissing potential oiling and treatment effects or interactions, we considered p ≤ 0.10 as potentially significant (when p > 0.05). SPSS statistical software (version 22) was used for all analyses. Our data are publically available on NOAA’s *Deepwater Horizon* Environmental Response Management Application (ERMA), http://gomex.erma.noaa.gov/erma.html (see the “NOAA Treatment Testing and Set-Asides Study” files under the “Response Sampling and Monitoring” folder).

### Ethics Statement

Authorization to conduct the treatment tests and study was provided by the *Deepwater Horizon* Unified Command (U.S. Coast Guard, State of Louisiana, and BP) and the Regional Response Team (RRT) under the Oil Pollution Act of 1990 and the *Deepwater Horizon* emergency response [2]. Operational shoreline treatments and maintenance and monitoring of the test plots and no treatment “set-asides” were authorized by STRs S3-045 and S4-032 (including variances and revisions) issued by the Unified Command, serving as both work plans and permits [2]. All subsequent research was conducted in coordination with the Unified Command and emergency response. The study area is privately owned; access permits were obtained from the landowner prior to conducting all work. No protected species were sampled. Invertebrate animals were held briefly in the field for identification and size measurements and were released unharmed into the areas from which they were collected at the completion of sampling. Efforts were made to minimize further habitat disturbance during sampling, mainly by using non-destructive methods, limiting the number of personnel and foot traffic in the marsh, and avoiding cross-contamination of sampling areas.

## Results and Discussion

### Pre-Treatment Oiling Conditions

**Fig 2:**
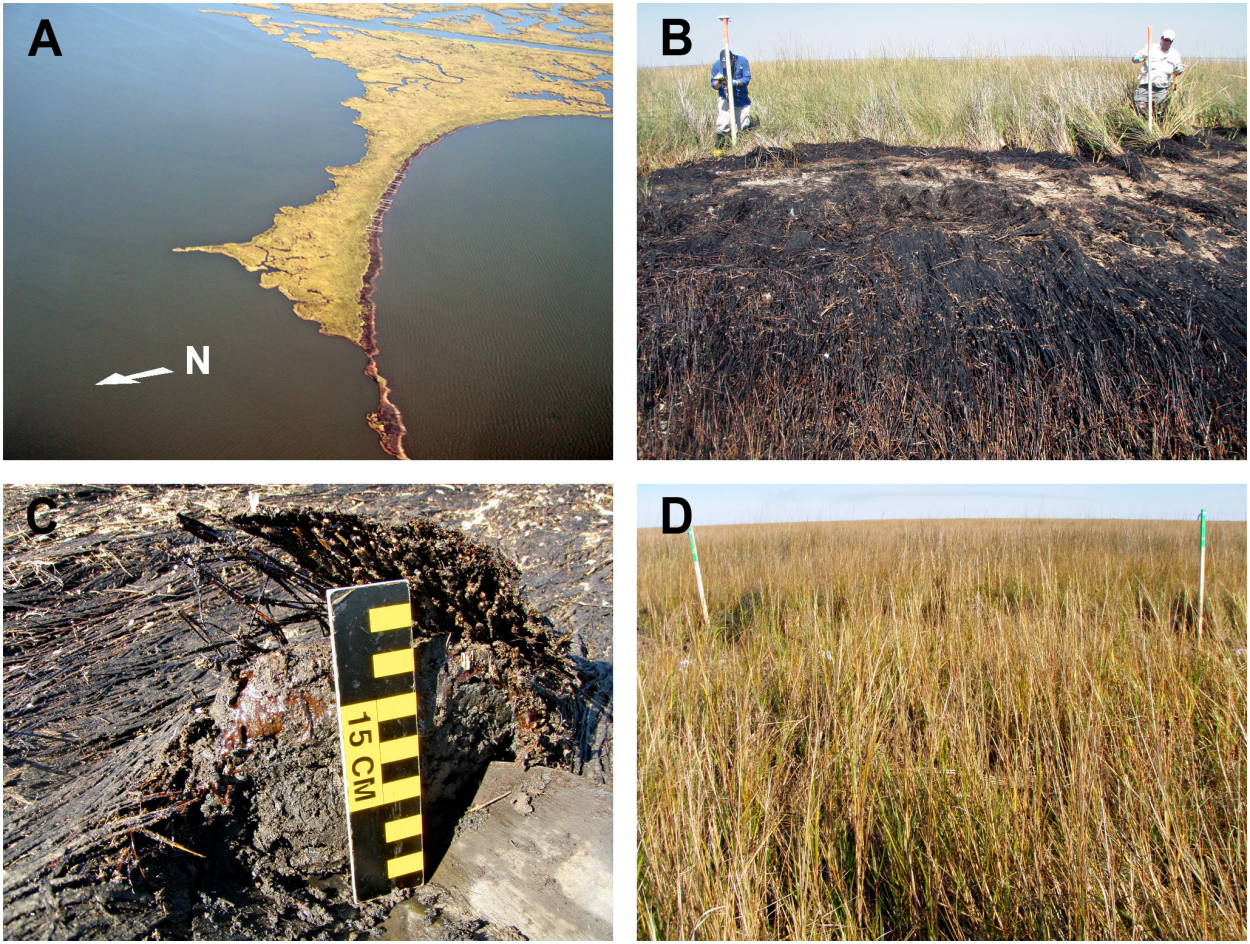
Pre-treatment oiling conditions. (A) Aerial view of heavy oiling distribution during plot set-up and adjacent reference area (shoreline at top right), (B) heavily oiled vegetation mat at plot-scale, (C) test pit depicting thick layer of emulsified oil (orange) on the marsh surface below the oiled vegetation mat, (D) reference plot for comparison.

Initial heavy oiling in northern Barataria Bay marshes occurred mainly in June 2010. The oil came ashore primarily as emulsified oil (an oil and water emulsion, also referred to as “mousse”) [2]. Floating oil and gross oiling on shorelines may have resulted in repetitive marsh oiling through August 2010, including the effects of tropical storms on oil remobilization and spreading. Pre-treatment oiling conditions were very similar and continuous across the heavily oiled test area (Fig 2 and S1 and S2 Figs, S1 Table, and following text). Oiling conditions were defined as two distinct oiling “zones” across the heavily oiled area. The first zone included a 1–3 m wide oiling band on the seaward marsh edge consisting of exposed surface oil residue (a solid, semi-cohesive mixture of oil and sediments) with 50–100% oil cover and ~1 cm oil thickness. The top layer of the surface oil residue had a dry, crusty consistency, including thin algal mats, a veneer of fine clay sediments, and surface cracking. All the vegetation in this zone died and sloughed off leaving only short vegetation stubble or no remnant vegetation.

The second oiling zone was contiguous with the first, extending further into the marsh. This zone included a 5–10 m wide band of heavily oiled vegetation mats and oiled wrack overlying a thick (>1 cm) layer of emulsified oil on the marsh substrate (Fig 2b and 2c). The oiled vegetation mats consisted of dead, laid over, and rooted marsh vegetation with a continuous oil coat of tarry consistency. Surviving marsh vegetation was absent or very limited in this zone. SCAT ground surveys conducted shortly after the oil came ashore mapped the heavily oiled test area as a single unit of comparable shoreline oiling with 95–100% surface oil cover. In late 2010, pre-treatment plot level assessments indicated 88% surface oil cover (and 87% oiled vegetation mat cover) across the heavily oiled plots, with no differences among treatment classes (S1 and S2 Figs). The emulsified surface oil layer had a typical thickness of 2–3 cm. Shoreline treatments focused on this second oiling zone, and consisted of removing the oiled vegetation mats and wrack and as much of the emulsified oil layer as possible, exposing the remaining surface oil to natural weathering and degradation processes, and minimizing the removal of marsh substrate.

The adjacent reference area was also oiled in part, but to a much lesser degree than the treatment test area. Vegetation in the reference area was not laid over or killed by the oil. As of December 2010, when the reference plots were established, the marsh vegetation had no visual signs of oiling and appeared dense and healthy at the seaward marsh edge and across all the reference plots. Two of the reference plots had no visible oiling on the marsh substrate, while three of the reference plots had substrate oiling consisting of small patches of surface oil residue at the seaward marsh edge. This oiling was ~0.5 m wide with 1–10% oil cover and ~0.3 cm oil thickness. No oiling was observed in the interior of the reference plots (there was no second oiling zone as described for the heavily oiled plots). The oiling levels observed in the reference plots during this study would be classified as “no oil observed” to light oiling according to SCAT methods [1].

### Post-Treatment Oiling Conditions

**Fig 3:**
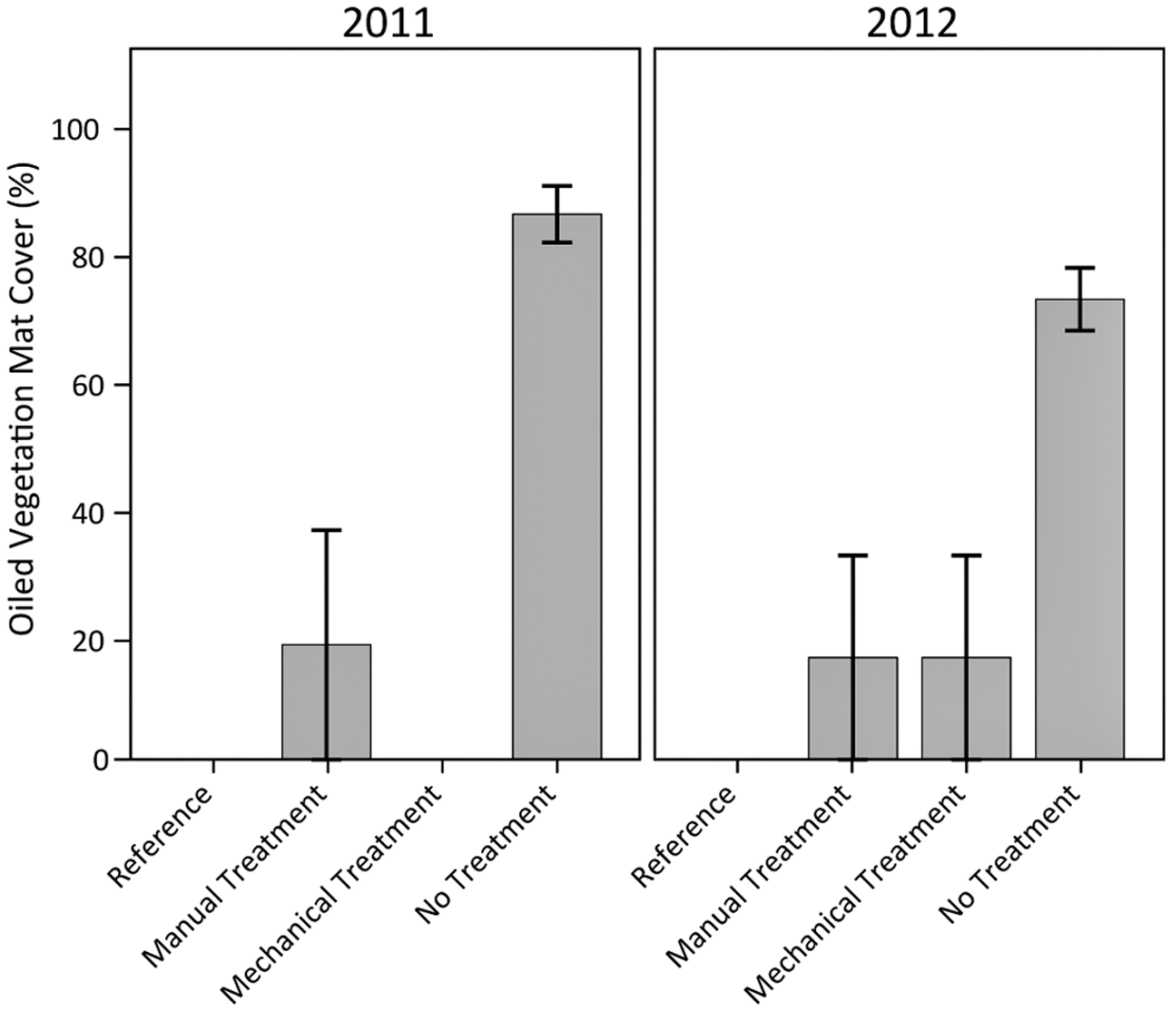
Oiled vegetation mat cover in 2011 and 2012. Differences among oiling/treatment classes were observed (p < 0.01); differences were not observed among years (p = 0.97); and potential differences were observed for the interaction of oiling/treatment class and year (p = 0.06). Specific oiling/treatment class differences were observed for no treatment versus all other classes (all p < 0.01). Data are means ± 1 standard error (SE). N = 9 for the heavily oiled plots with no treatment; n = 5 for all other oiling/treatment classes including reference.

Oiled vegetation mat cover in the no treatment plots remained relatively high and distinct from the treated plots which had little to no mat presence following treatment (Fig 3, S1 Table). One plot each for the manual and mechanical treatments had a narrow zone of oiled vegetation mat near the landward extent of the plot that was missed during treatment because it was buried and not visible. These areas were subsequently unburied by natural processes, and so were visible and recorded during the next sampling event, explaining the apparent “increase” in oiled vegetation mat cover for the mechanical treatment plots in 2012 (a mat was buried and not visible in 2011, but became unburied and was observed in 2012).

**Fig 4:**
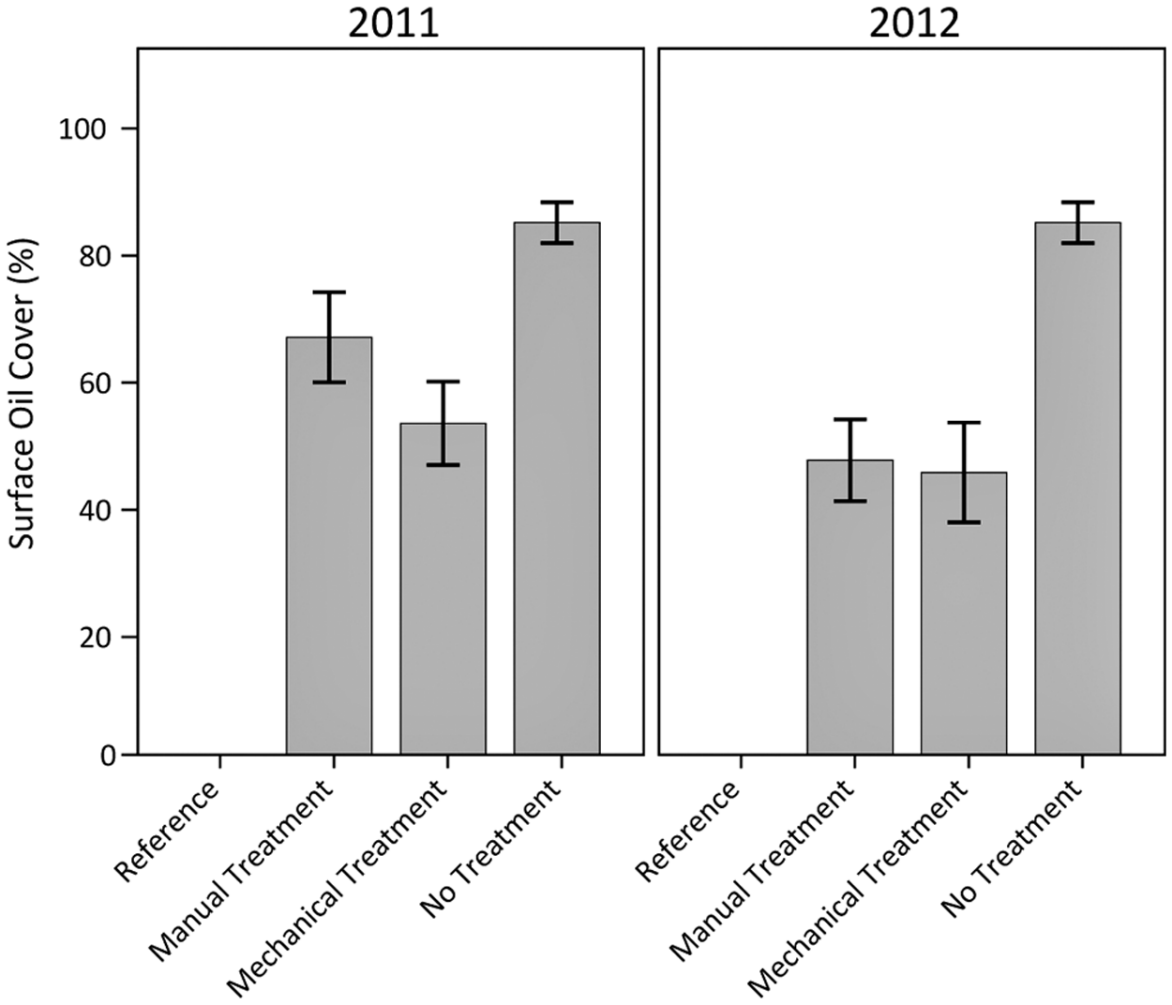
Surface oil cover in 2011 and 2012. Differences among oiling/treatment classes (p < 0.01) and years (p = 0.03) were observed; potential differences were observed for the interaction of oiling/treatment class and year (p = 0.09). Specific oiling/treatment class differences were observed for reference versus all other classes, and manual and mechanical treatment versus no treatment (all p < 0.01). Data are means ± 1 SE. N = 9 for the heavily oiled plots with no treatment; n = 5 for all other oiling/treatment classes including reference.

Oil cover on the soil surface remained well above reference values for the heavily oiled plots, regardless of treatment (Fig 4). The manual and mechanical treatment plots had similar surface oil cover, and both had less surface oil cover than the no treatment plots, indicating an improvement in surface oiling conditions with treatment. In addition, both manual and mechanical treatments converted the dominant oiling character in 100% of the treated plots from emulsified oil to a more weathered surface oil residue, whereas the primary surface oiling type in all the no treatment plots continued to be emulsified oil, remaining unchanged through 2012. The time difference between manual and mechanical treatment did not appear to affect surface oiling characteristics, probably due to the heavy and persistent oiling conditions and slow natural degradation processes associated with thick oiling and wetland soils.

There were no observed signs of increased oil penetration or mixing of oil into the subsurface soil as a result of manual treatment. However, signs of increased subsurface oiling caused by mechanical treatments were observed in the test pits. In most of the mechanical treatment plots, heavy oil residues or emulsified oil were mixed into the soil to depths of 5–20 cm. Mixing of oil into marsh soils during treatment is typically viewed as detrimental, because subsurface oiling weathers and degrades more slowly than surface oiling, resulting in longer-term contamination which may chronically affect marsh vegetation and infauna [5].

**Fig 5:**
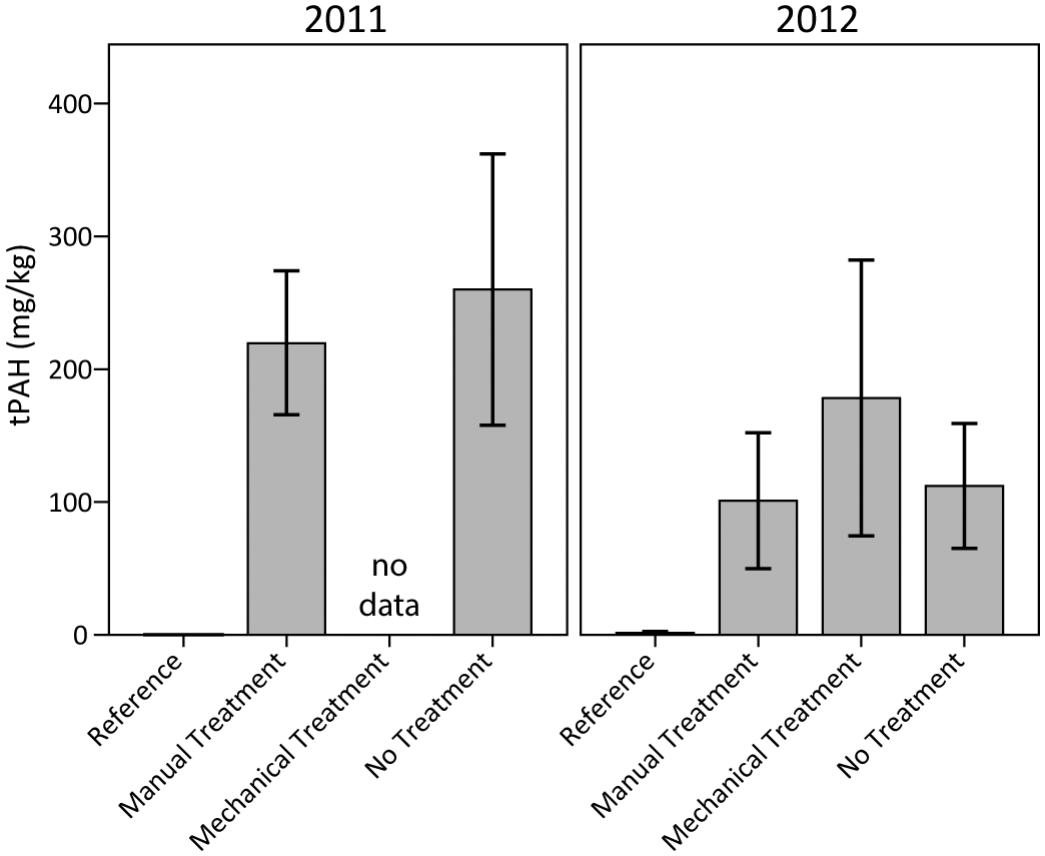
Total polycyclic aromatic hydrocarbons (tPAH) in subsurface marsh soils in 2011 and 2012. Differences among oiling/treatment classes were observed (p = 0.04); differences were not observed among years (p = 0.17) or for the interaction of oiling/treatment class and year (p = 0.59). Specific oiling/treatment class differences were observed for reference versus no treatment (p = 0.05; reference versus manual treatment was p = 0.16). Data are means ± 1 SE. N = 9 for the heavily oiled plots with no treatment; n = 5 for all other oiling/treatment classes including reference. Due to lack of data in 2011, the repeated measures ANOVA excluded mechanical treatment. An ANOVA was applied to the 2012 data including mechanical treatment; differences were not significant (p = 0.32).

Subsurface tPAH concentrations in the marsh soils generally indicated differences between the reference plots and the heavily oiled plots, though differences were only statistically significant for reference versus no treatment (Fig 5). The chemistry results validate the low level of background oiling in the reference plots versus the heavy oiling conditions in the other plots. Although the manual and mechanical treatments improved surface oiling conditions as described above, they did not substantially change tPAH concentrations in the subsurface soils relative to the no treatment plots. On the other hand, these results also indicate that treatments did not worsen oiling levels in the subsurface soils, one of the primary concerns during marsh treatment. This agrees with visual observations that the manual treatments did not mix oil into the soils; however, it conflicts with the direct observations that mechanical treatment did mix oil into the soils. These results may partly reflect the variable distribution of oil in marsh soils, as well as the limitations of soil grab samples and likely insufficient sampling, making it difficult to detect potential differences among treatments. Given similar circumstances, we would suggest taking additional soil samples from each plot to better address tPAH concentrations, as well as conducting targeted sampling of oiled intervals within test pits used to describe subsurface oiling.

**Fig 6:**
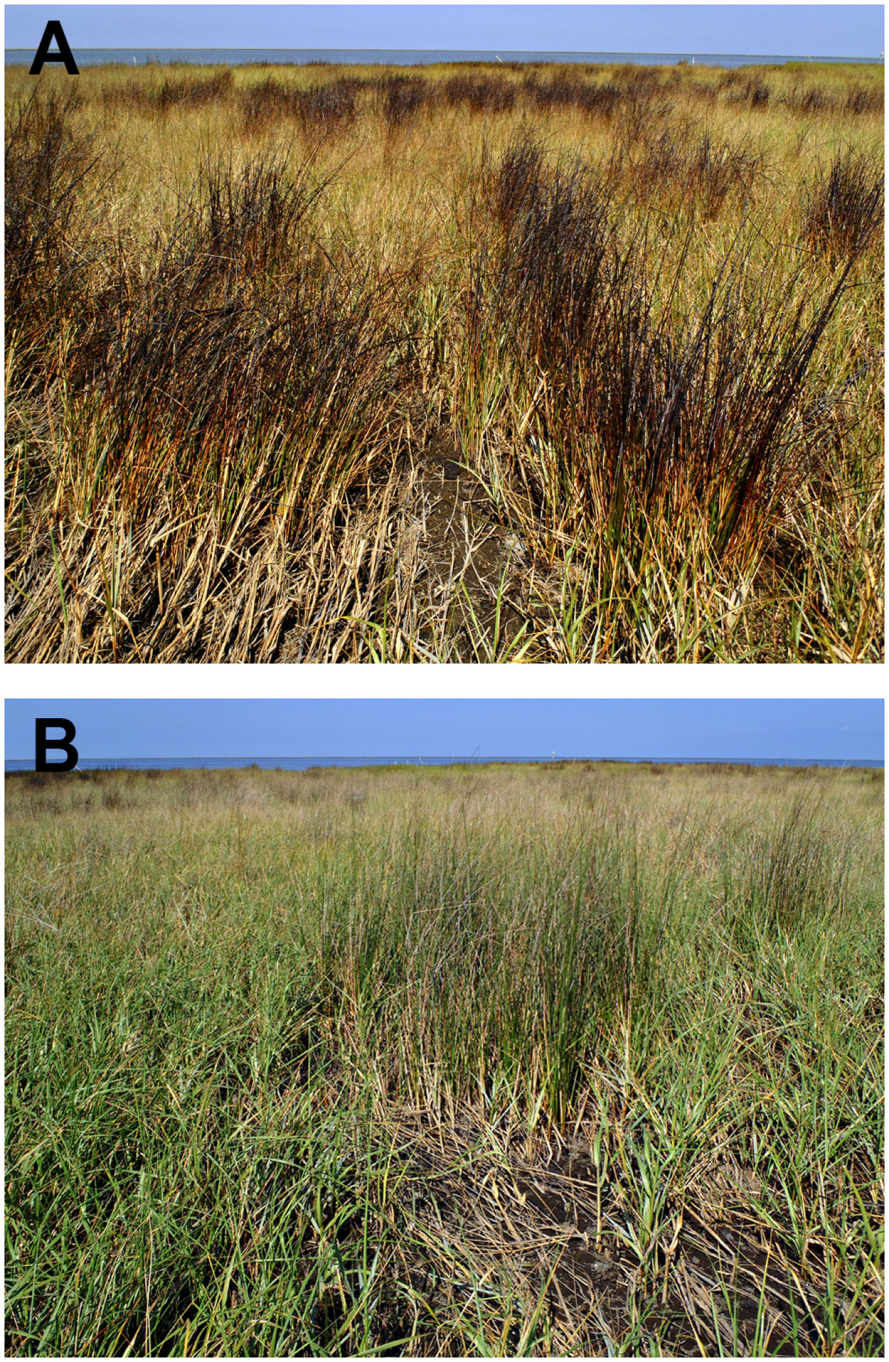
Example of differences in localized oil remobilization following Tropical Storm Lee in 2011. (A) Remobilized oil on marsh vegetation extending > 50 m landward (inland) of a no treatment plot, (B) absence of remobilized oil landward of the adjacent manual treatment plot.

Severe storms in late April 2011, Tropical Storm Lee in early September 2011, and Hurricane Isaac in late August 2012 caused localized re-mobilization of oil from the existing oiled marsh in the study area (meaning oil already in the marsh was mobilized and locally redistributed during these storms). These events resulted in oiling of new vegetation growth within the plots, as well as oiling of previously unoiled vegetation landward of the plots (to a few meters beyond the plots in April 2011; to tens of meters beyond the plots during Tropical Storm Lee; and to several meters beyond the plots during Hurricane Isaac). In all cases, oil remobilization was noticeably less in areas that were treated, and there was no remobilized oil observed in the reference area. Following Tropical Storm Lee in particular, there was a marked difference in the degree of oil re-mobilization from the manual and mechanical treatment areas (with little to no oil remobilization) compared to the no treatment plots (with substantial oil remobilization) (Fig 6).

Overall, both the manual and mechanical treatments were effective in removing the heavily oiled vegetation mats and wrack, reducing the degree of surface oiling, converting the dominant surface oiling condition from emulsified oil to a more weathered surface oil residue, and reducing oil remobilization. In contrast, oiling conditions in the no treatment plots remained largely unchanged throughout the study. Though still differing substantially from reference conditions, both manual and mechanical treatments improved surface oiling conditions considerably. However, mechanical treatment also resulted in visible mixing of oil into the marsh soils, which is typically detrimental to marsh recovery.

### Vegetation

**Fig 7:**
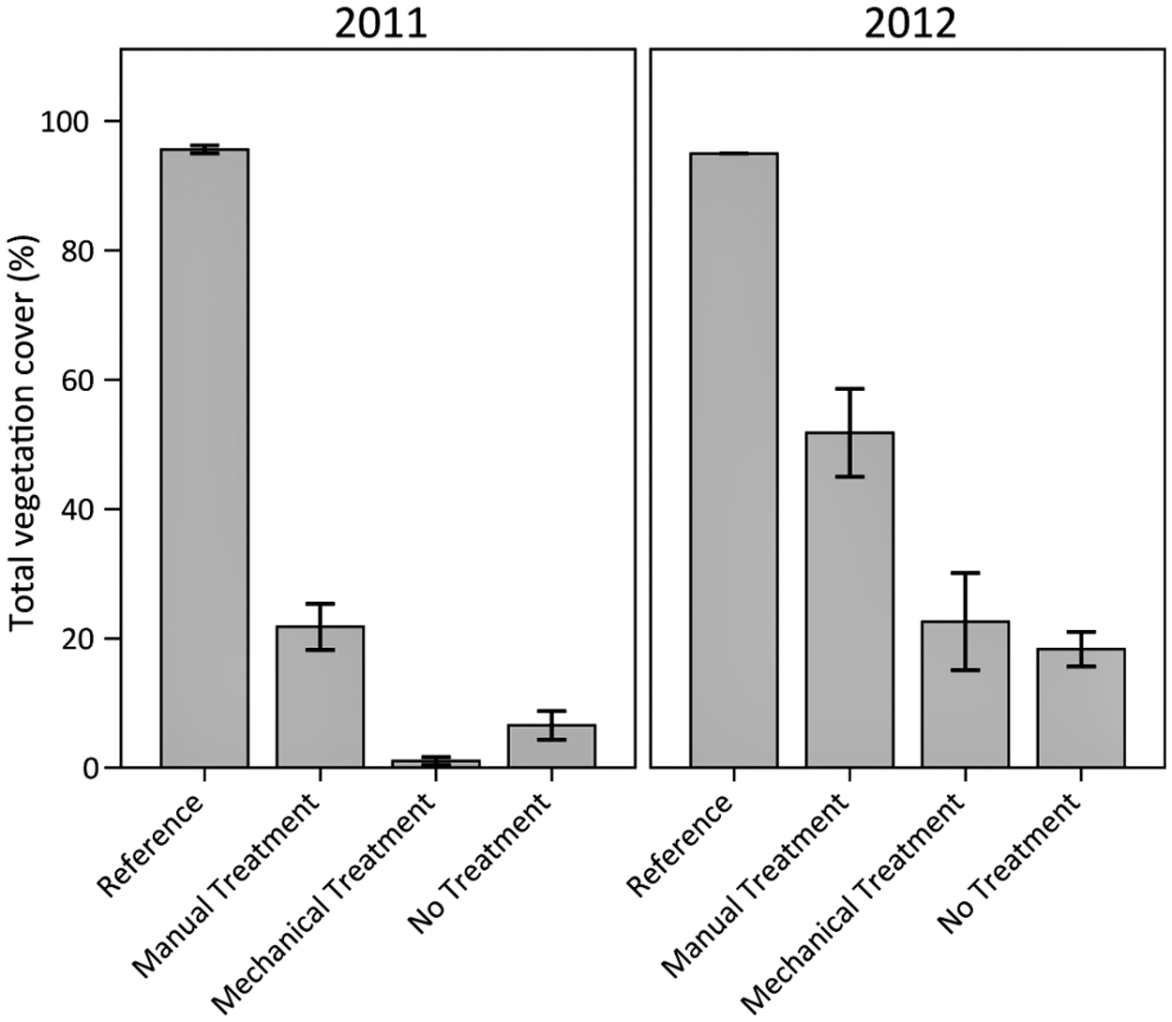
Total vegetation cover in 2011 and 2012. Differences among oiling/treatment classes, years, and the interaction of oiling/treatment class and year were observed (all p < 0.01). Specific oiling/treatment class differences were observed for reference versus all other classes, and manual treatment versus mechanical treatment and no treatment (all p < 0.01). Data are means ± 1 SE. N = 9 for the heavily oiled plots with no treatment; n = 5 for all other oiling/treatment classes including reference.

Total vegetation cover in the heavily oiled plots was well below reference values regardless of treatment and year (Fig 7), indicating that vegetation cover had not recovered more than two years following initial heavy oiling. Mechanical treatment took place in 2011, explaining the very low cover values recorded in that year. Among the heavily oiled plots, manual treatment had the greatest vegetation cover in both years, exceeding 50% of reference values in 2012. Mechanical treatment values in 2012 were similar to those for manual treatment in 2011, both approximately one year post-treatment; therefore, these treatments may be somewhat comparable in terms of vegetation cover, at least through one year. All the heavily oiled plots showed increases in vegetation cover with time, whether treated or untreated, indicating some initial vegetation recovery in progress; however, untreated values were still quite low more than two years following initial oiling. Overall, active treatments appeared to have a positive influence on total vegetation cover compared to no treatment; this was particularly evident for manual treatment, though recovery of total vegetation cover was still incomplete.

**Fig 8:**
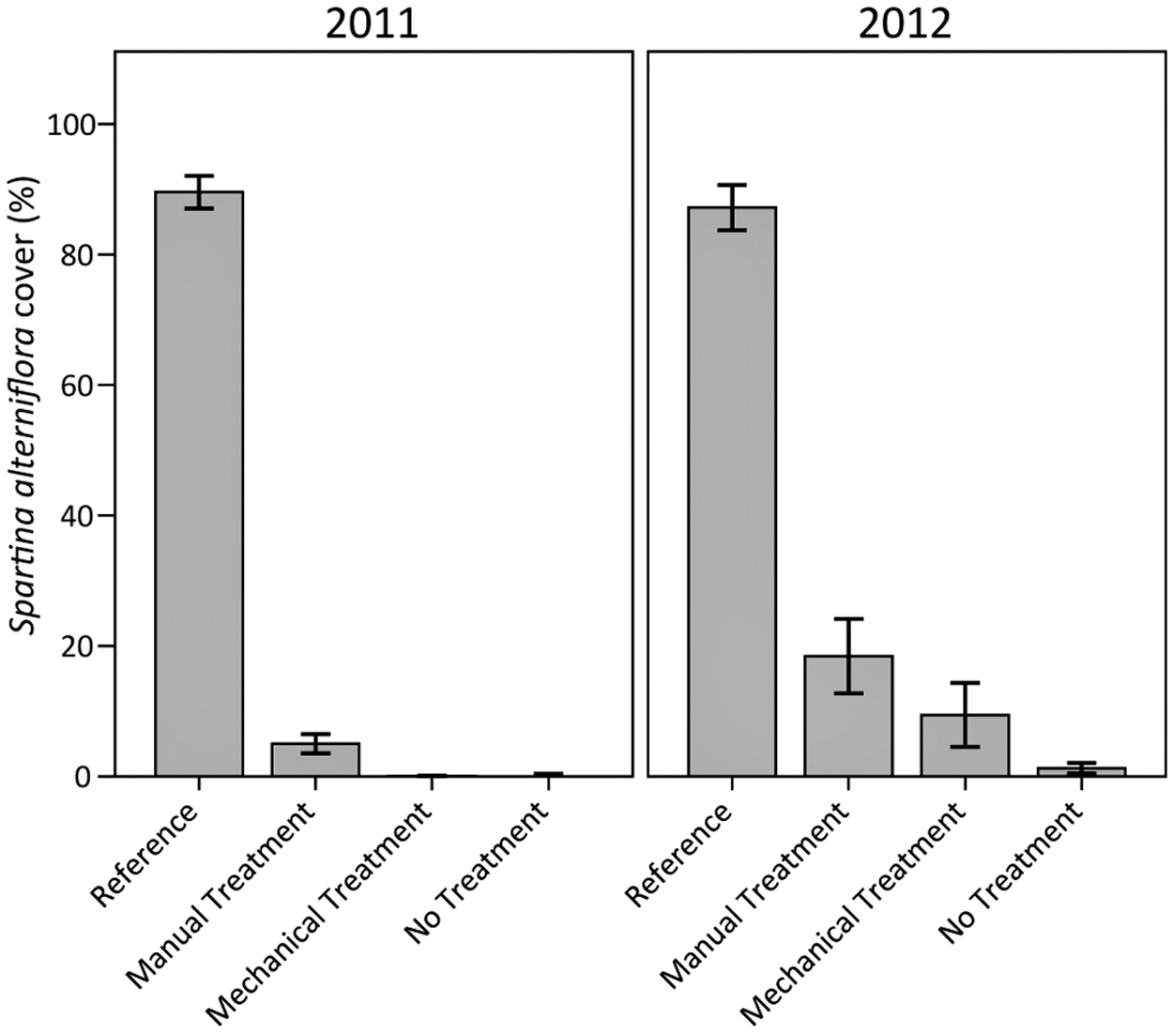
*Spartina alterniflora* cover in 2011 and 2012. Differences among oiling/treatment classes (p < 0.01), years (p < 0.01), and the interaction of oiling/treatment class and year (p = 0.01) were observed. Specific oiling/treatment class differences were observed for reference versus all other classes, and manual treatment versus no treatment (all p < 0.01). Data are means ± 1 SE. N = 9 for the heavily oiled plots with no treatment; n = 5 for all other oiling/treatment classes including reference.

*S*. *alterniflora* is the dominant species of salt marsh vegetation in the study area, as evidenced by cover values from the reference plots (Figs 7 and 8). The pattern in *S*. *alterniflora* cover among plots was similar to total vegetation cover, although *S*. *alterniflora* cover values were much lower across the heavily oiled plots compared to reference conditions. Among the heavily oiled plots, *S*. *alterniflora* cover was greatest in the manual treatment plots in both years, though not statistically different from the mechanical treatment plots. By 2012, *S*. *alterniflora* cover slightly exceeded 10% and 20% of reference values for the plots with mechanical and manual treatments, respectively. *S*. *alterniflora* cover in the no treatment plots was very low in both years (0–1%), showing little recovery more than two years following initial oiling, similar to the findings of Lin and Mendelssohn (2012) [26] at seven months following heavy oiling. Active treatment had a positive influence on *S*. *alterniflora* cover, but not to the same extent observed for total vegetation cover, indicating species composition differences within the recovering vegetation. In addition, these results suggest vegetation recovery may be ongoing for several years with treatment and even longer without treatment [5,15].

**Fig 9:**
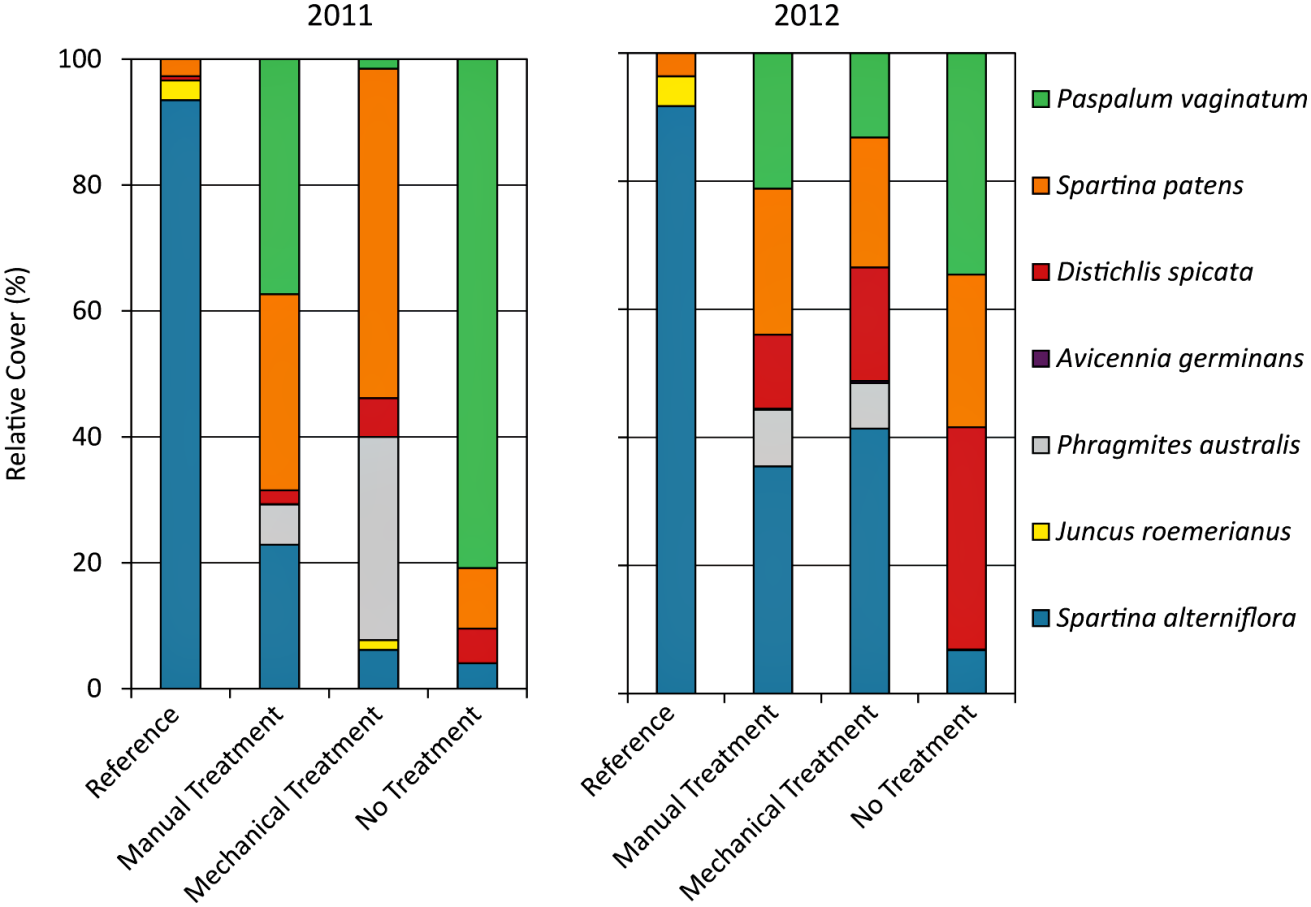
Vegetation species composition in 2011 and 2012.

The reference plots were strongly dominated by *S*. *alterniflora* in both years, with smaller contributions by *J*. *roemerianus*, *S*. *patens*, and *Distichlis spicata* (Fig 9), typical for salt marshes in the region [43–44]. *Avicennia germinans* (black mangrove) shrubs and seedlings occurred sporadically in the reference area, but were not recorded in the reference plots. The heavily oiled plots were quite different, whether treated or not, with dominance shared among several species, including *S*. *patens*, *Paspalum vaginatum*, *Phragmites australis*, *D*. *spicata*, and *S*. *alterniflora*. Both *S*. *alterniflora* and *J*. *roemerianus* were originally present and appeared to be dominant prior to the spill in the heavily oiled plots, based on the appearance of the oiled vegetation mats and the intact vegetation landward of the plots (based on initial field observations and review of plot photos). Therefore, the pre-spill vegetation composition in the heavily oiled plots was similar to the adjacent reference area. The observed difference in marsh species composition in the heavily oiled plots may have been a result of several factors: the nearly complete vegetation dieback resulting from heavy oiling; differing sensitivities and reactions to oiling and disturbance among plant species; and initial plant re-colonization during lower salinities associated with freshwater diversion enacted in reaction to the spill [45].

In 2011, the main comparison of vegetation composition was between the manual treatment plots and the no treatment plots, since the mechanically treated plots had recently been treated and contained little vegetation. Though the species recorded were similar, the manual treatment plots had a more even distribution of species and greater relative cover of *S*. *alterniflora* and *S*. *patens* as compared to the no treatment plots, which were strongly dominated by *P*. *vaginatum*. The manual treatment plots also had recruitment of *A*. *germinans* seedlings into the plots following treatment. Small patches of *P*. *australis* were already present in some plots and survived oiling by re-sprouting from rhizomes, but did not spread or further colonize the marsh following oiling or treatment. In contrast, all the other species in the heavily oiled plots either expanded into the plots from outside or newly colonized the plots following oiling or treatment.

The occurrence of *P*. *vaginatum* in the plots, particularly its heavy dominance in the no treatment plots, was unexpected, as this species is more characteristic of lower salinity coastal marsh rather than salt marsh [43–44]. However, *P*. *vaginatum* can be a disturbance-associated species [46–48], which may explain its presence in the heavily oiled plots, especially in combination with a period of lower salinity. *P*. *vaginatum* was often the first species to appear in the plots and was observed colonizing directly on top of the heavily oiled vegetation mats. *P*. *vaginatum* may have been reduced in the manual and mechanical treatment plots by the removal of the oiled vegetation mats and direct raking and cutting of *P*. *vaginatum*, perhaps enhancing the later colonization or spreading of other species. Overall, as of 2011, manual treatment appeared to result in vegetation composition more similar to reference conditions as compared to no treatment.

In 2012, both the manual and mechanical treatment plots were similar in species composition and trending more towards reference conditions, with increasing relative cover of *S*. *alterniflora*. *J*. *roemerianus* was not present in any of the heavily oiled plots in 2012. Impacts and lack of *J*. *roemerianus* recovery in heavily oiled areas is consistent with other studies [26]. *J*. *roemerianus* generally has low tolerance to oiling and is one of the slowest species to recover from oiling or disturbance in general [26,49–50]. The no treatment plots were also less dominated by *P*. *vaginatum* in 2012, though still more so than in the treated plots. The no treatment plots also had a more even distribution of species in 2012, including *S*. *patens* and increased relative cover of *D*. *spicata;* though still with relatively little *S*. *alterniflora*. Increases in relative cover of *D*. *spicata* in the heavily oiled plots may have been related to increasing salinity once freshwater diversion was scaled back. *D*. *spicata* is also frequently associated with disturbance in salt marshes [51]. Other than the lack of *J*. *roemerianus* recovery [26], changes in vegetation species composition in salt marshes in relation to *Deepwater Horizon* impacts have not been reported elsewhere and may warrant further investigation. Changes in vegetation composition could have a variety of ecosystem implications, ranging from changes in microbial assemblages and faunal consumers to decreased marsh stability. Overall, through 2012, though still quite different from reference conditions, both the manual and mechanical treatments resulted in vegetation composition more similar to reference conditions compared to no treatment.

### Marsh Periwinkles

**Fig 10:**
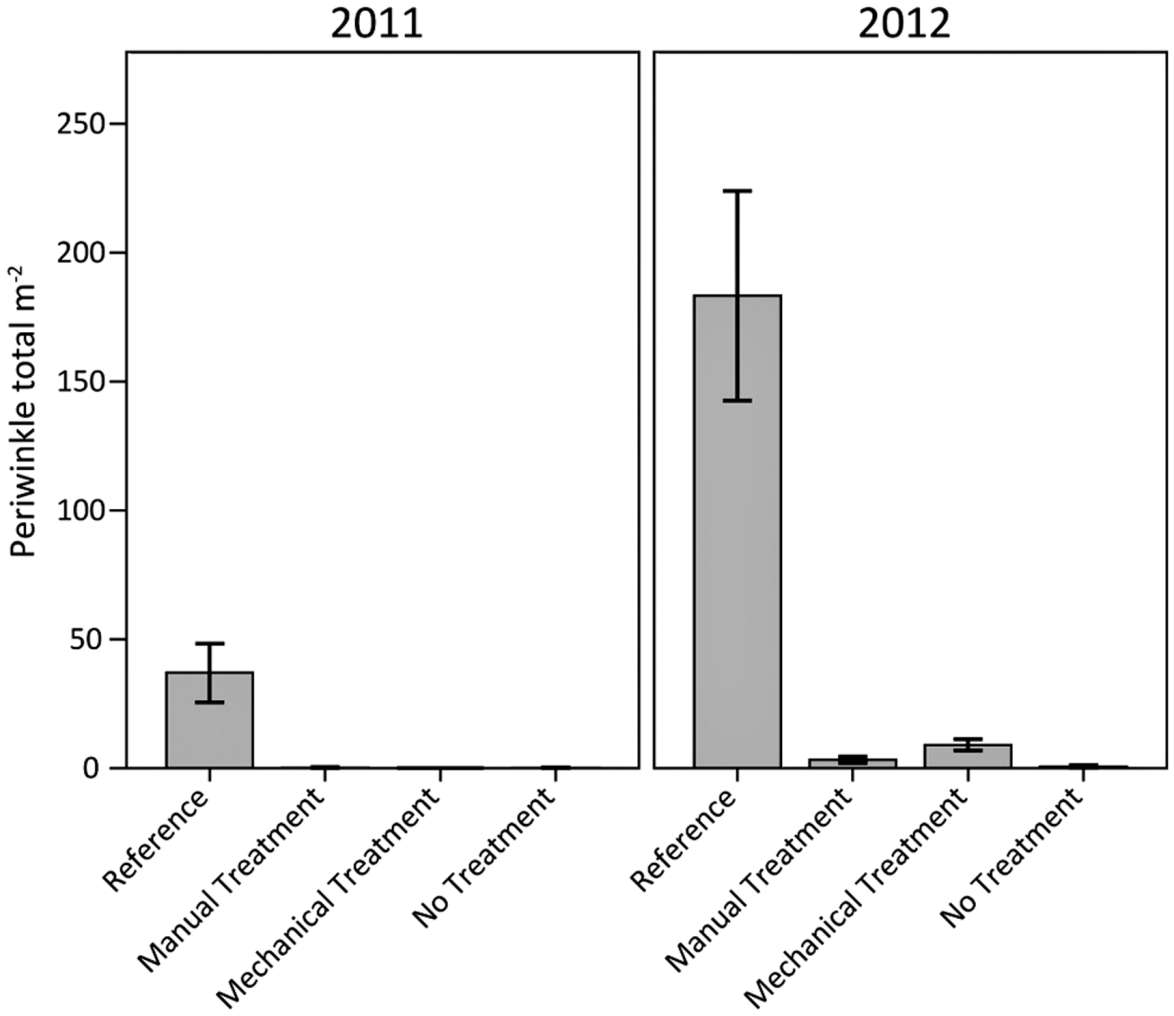
Total marsh periwinkle (*Littoraria irrorata*) densities in 2011 and 2012. Differences among oiling/treatment classes, years, and the interaction of oiling/treatment class and year were observed (all p < 0.01). Specific oiling/treatment class differences were observed for reference versus all other classes (p < 0.01). Data are means ± 1 SE. N = 9 for the heavily oiled plots with no treatment; n = 5 for all other oiling/treatment classes including reference.

Total marsh periwinkle (*Littoraria irrorata*) densities in the heavily oiled plots were well below reference values regardless of treatment and year (Fig 10). Marsh periwinkles were nearly absent from the heavily oiled plots in 2011. In 2012, there were very low numbers of snails in the heavily oiled plots, <1–5% of reference values, indicating minimal periwinkle recovery. These results are similar to an earlier *Deepwater Horizon* study from heavily oiled sites in 2010 [28], with our study indicating continuing marsh periwinkle impacts through 2012, more than two years following heavy oiling. In a related ongoing *Deepwater Horizon* study, we reported periwinkle impacts extending through 2013 (more than three years following heavy oiling), with possible initial signs of recovery limited to sites planted with *S*. *alterniflora* [52].

**Fig 11:**
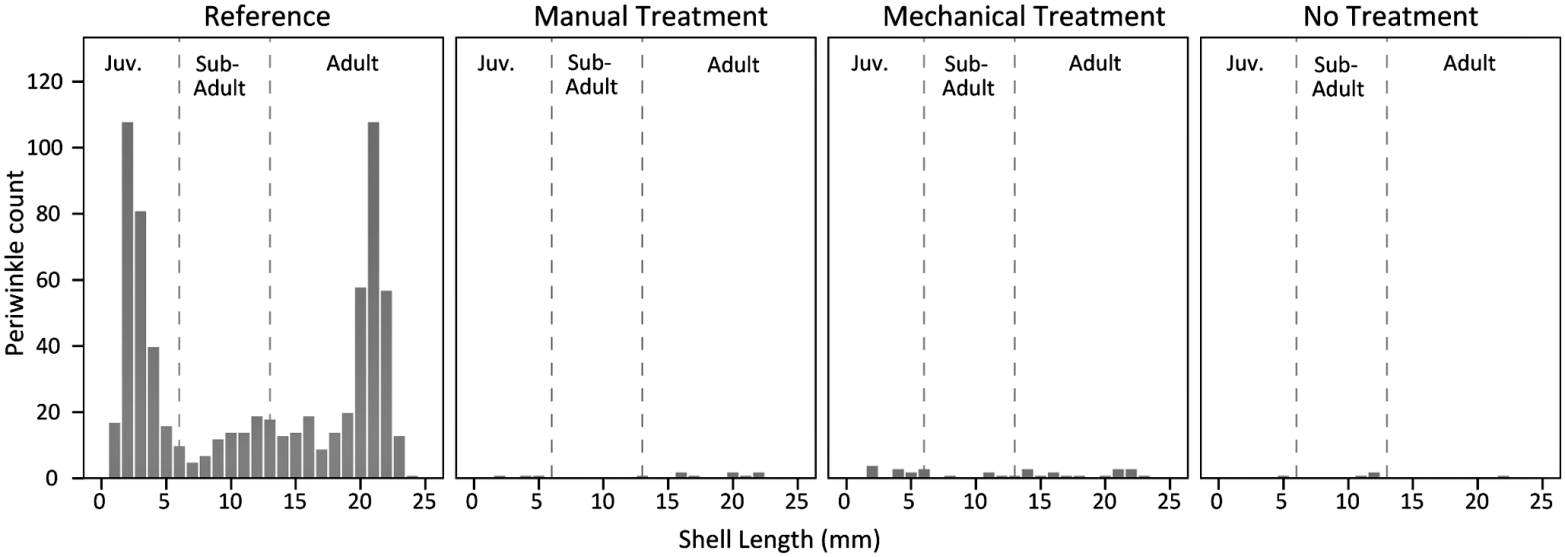
Marsh periwinkle (*Littoraria irrorata*) shell length frequencies in 2012. Periwinkle life-history size ranges defined as juveniles (<6 mm), sub-adults (6–13 mm), and adults (>13 mm). Juvenile, sub-adult, and adult snail densities were greater for the reference plots versus all other classes (all p ≤ 0.01).

Snail size frequency histograms from 2012 for the reference plots depicted a bimodal to tri-modal pattern with two major peaks, one for juveniles and one for adults, and a smaller peak encompassing sub-adults (Fig 11). We think this may be a typical size distribution pattern for sites with established adult periwinkle populations, juvenile recruitment in the prior year (leading to sub-adults), and recent juvenile recruitment [17,42,53–54]. In contrast to the reference plots, the heavily oiled plots had low numbers of snails across all sizes and life stages. Juveniles were largely absent from the heavily oiled plots in 2012, indicating either a lack of juvenile recruitment or limited survival. The near absence of juveniles as well as sub-adults further indicates that periwinkle recovery in the heavily oiled plots was minimal to absent by 2012.

Marsh periwinkles are closely associated with the dominant salt marsh vegetation, particularly *S*. *alterniflora*. For example, Kiehn and Morris (2009) [55] reported that marsh periwinkle presence and density were positively correlated with *S*. *alterniflora* stem density. Similarly, Stagg and Mendelssohn (2012) [41] found that marsh periwinkle growth, survival, and productivity were positively correlated with *S*. *alterniflora* cover in a sediment-restored salt marsh in Louisiana. Therefore, recovery of marsh periwinkles in the heavily oiled plots may be tied to the recovery of *S*. *alterniflora*, though residual oiling levels may also be a factor. Marsh periwinkle recovery, including density and population structure, may lag *S*. *alterniflora* recovery for several years once conditions are suitable to support recruitment, immigration, survival, and growth. Lags in recovery or development of marsh periwinkle densities and population structure relative to vegetation conditions have been observed following oil spills [16–17], physical marsh impacts [56], and marsh restoration and creation projects [57–58]. Further study is needed concerning marsh periwinkle impacts and recovery, and the potential positive effects of planting *S*. *alterniflora* in heavily oiled areas.

### Fiddler Crabs

**Fig 12:**
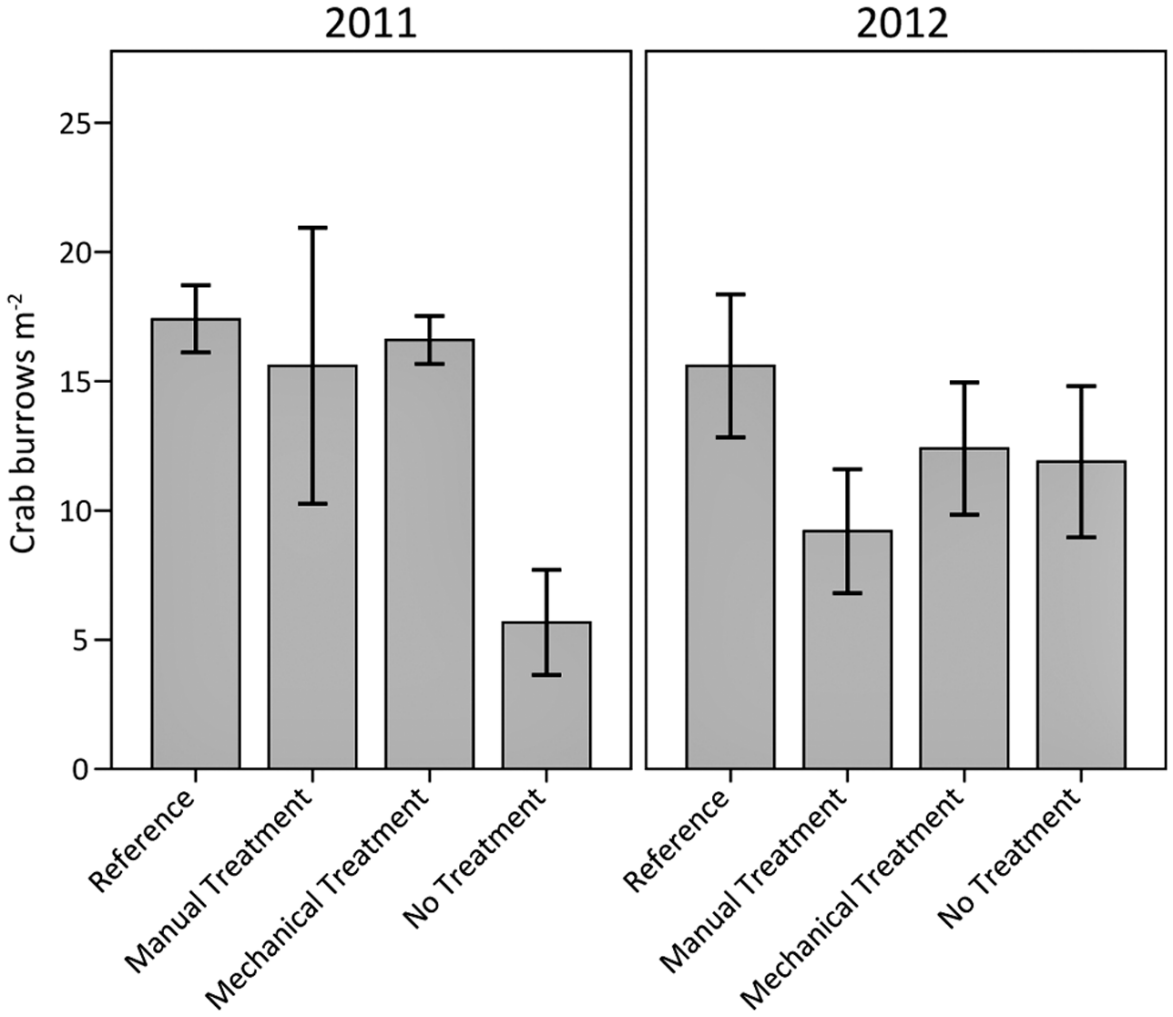
Crab burrow densities (*Uca* spp.) in 2011 and 2012. Differences among oiling/treatment classes were observed (p = 0.05); differences were not observed among years (p = 0.47) or for the interaction of oiling/treatment class and year (p = 0.12). Potential differences among specific oiling/treatment classes were observed for reference versus no treatment (p = 0.06). Data are means ± 1 SE. N = 9 for the heavily oiled plots with no treatment; n = 5 for all other oiling/treatment classes including reference.

Crab burrow densities (mainly fiddler crabs, *Uca* spp.) in the heavily oiled and treated plots were similar to reference values. However, in the heavily oiled plots without treatment, burrow densities were lower than reference conditions (mainly in 2011) (Fig 12). At the time of sampling in 2011, crab burrow densities in the manual and mechanical treatment plots were similar to reference levels. By 2012, the no treatment plots were similar to reference levels as well. Treatment may have hastened the return of crab burrow densities by about one year. The removal of the heavily oiled vegetation mats and the reduction of the thick emulsified oil layer, as well as raking of the underlying substrate, may have facilitated fiddler crab recruitment and immigration from adjacent areas.

Impacts and recovery of fiddler crabs were reported in a previous *Deepwater Horizon* study [27]. Our study shows density reductions in areas with heavier oiling and without treatment extending for an additional year into 2011. Impacts to fiddler crabs have also been observed at prior spills. Most notably, fiddler crab impacts following the *Florida* barge spill in Buzzards Bay, Massachusetts were still evident 37 years later due to long-term sediment contamination (impacts included reduced burrow densities) [19]. The *Florida* spill was different from the *Deepwater Horizon* spill in the following ways: the spilled material was a No. 2 fuel oil, which generally has greater chemical toxicity than most crude oils [5]; the *Florida* spill occurred much closer to shore and near the water surface, with less time for oil weathering before making landfall; and the *Florida* spill occurred in a colder climate, perhaps slowing oil weathering and degradation rates, as well as affecting fiddler crab behavior and impacts (e.g., crabs needing to shelter in burrows during low winter temperatures in contaminated soils—see [20–21]). These differences could explain why fiddler crab burrow density reductions were not as long-term in our study, at least based on sampling through 2012. However, in a related ongoing *Deepwater Horizon* study, we reported possible continuing impacts to fiddler crab burrow density into 2013 with a potential positive influence of *S*. *alterniflora* planting in oiled and treated plots [52].

**Fig 13:**
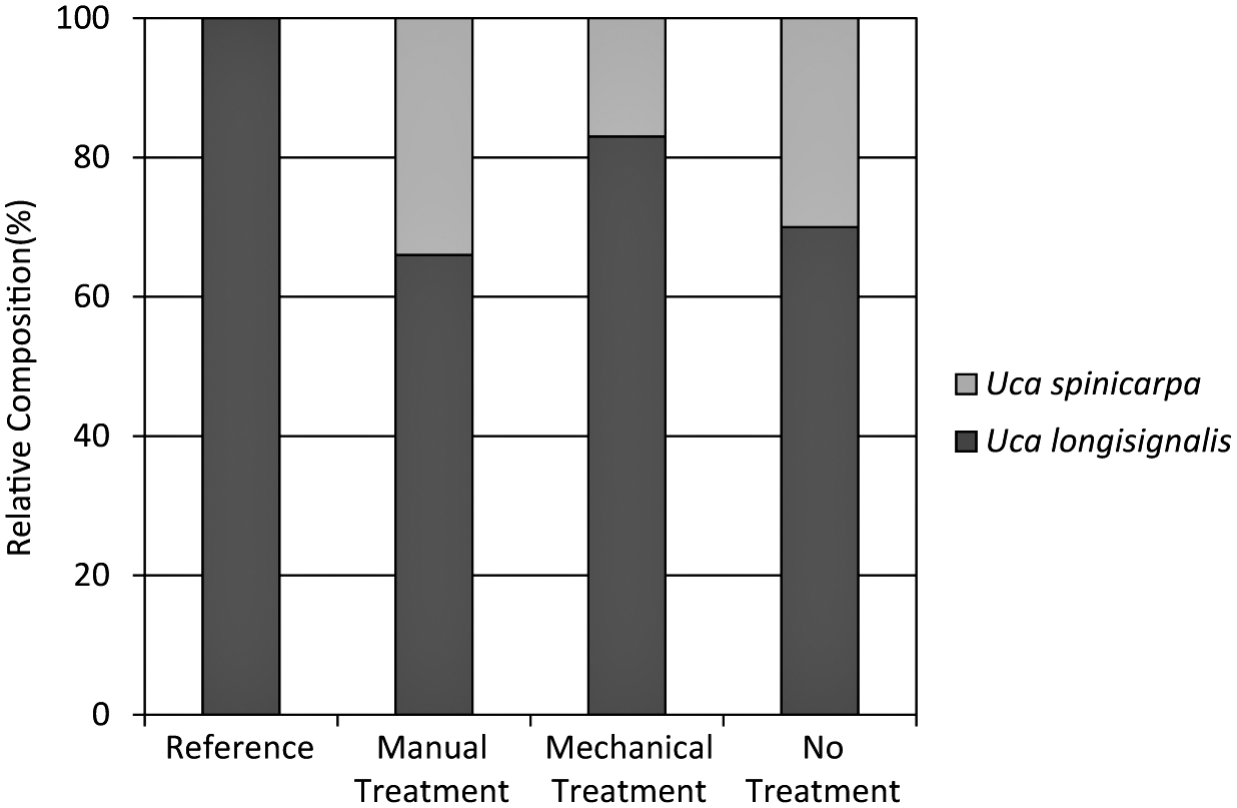
Fiddler crab (*Uca* spp.) relative species composition in 2012.

In contrast to burrow density in the present study, fiddler crab species composition may point to continuing differences among the reference and heavily oiled plots (Fig 13). *Uca longisignalis* was 100% dominant in the reference plots, whereas the heavily oiled plots had relative compositions of 66–83% *U*. *longisignalis* and 17–34% *Uca spinicarpa*. Oiling and associated habitat alterations may have led to these differences. *U*. *longisignalis* typically dominates densely vegetated salt marsh sites in Louisiana, whereas *U*. *spinicarpa* dominates clay banks with sparse vegetation [59]. Where the two species co-occur in salt marsh sites dominated by *U*. *longisignalis*, *U*. *spinicarpa* is typically restricted to the seaward marsh edge and may comprise ≤10% of the population [59]. Reduced vegetation cover coupled with areas of surface oil residue overlaid with thin algal mats and clay sediments may have led to greater relative abundance of *U*. *spinicarpa* in the heavily oiled sites. During sampling, *U*. *longisignalis* was typically captured in vegetated portions of the plots; while *U*. *spinicarpa* was typically captured in sparsely vegetated areas with surface oil residue. In a related, ongoing *Deepwater Horizon* study, we reported similar differences in species composition in 2013; however; *S*. *alterniflora* planting in oiled and treated plots resulted in 100% dominance by *U*. *longisignalis*, matching reference conditions [52]. We are not aware of any *Deepwater Horizon* or other oil spill studies that have examined changes in fiddler crab species composition. Further study is needed concerning fiddler crab impacts and recovery in relation to oiling, shoreline treatment, and planting, including the causes and effects of species composition changes. Similar to marsh periwinkles, fiddler crab recovery may also be linked to continuing vegetation recovery.

### Marsh Shoreline Retreat

**Fig 14:**
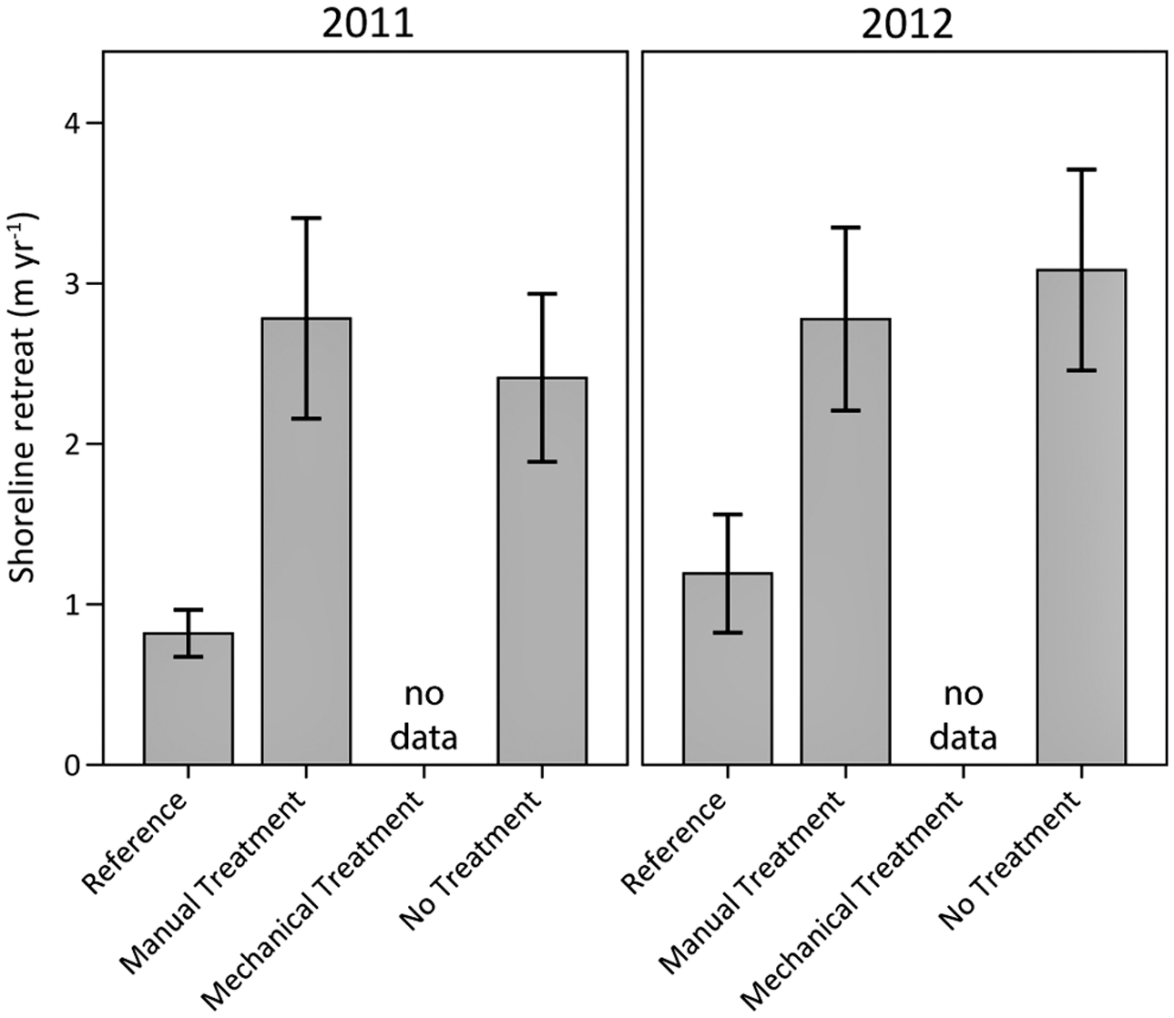
Marsh shoreline retreat in 2011 and 2012. Differences among oiling/treatment classes were observed (p < 0.01); differences were not observed among years (p = 0.58) or for the interaction of oiling/treatment class and year (p = 0.89). Specific oiling/treatment class differences were observed for reference versus manual treatment and no treatment (both p < 0.01). Data are means ± 1 SE. N = 9 for the heavily oiled plots with no treatment; n = 5 for all other oiling/treatment classes including reference.

The rate of marsh shoreline retreat was 2–3 times greater in the heavily oiled plots compared to the reference plots in 2011 and 2012, whether manually treated or not treated (Fig 14). Directly comparable erosion data were not collected for the mechanical treatment plots under the main study. However, qualitatively, it did appear that the mechanical treatments lowered the marsh surface and that these areas were experiencing even greater erosion (see following section).

Shoreline retreat rates in the reference plots were very similar to previously published rates of 0.8–1.3 m yr^-1^ for this portion of Barataria Bay [60] (based on [61]), indicating that the reference plots were within the range of expected background erosion rates. The greater shoreline retreat in the heavily oiled plots was likely due to vegetation die-back and subsequent soil weakening as a result of the oil spill and incomplete vegetation recovery more than two years later (Figs 7 and 8). Increased marsh erosion has been reported following other oil spills [62–65]. In addition, our results are similar to those observed in prior *Deepwater Horizon* studies [28–29], with the exception that we observed accelerated erosion during both 2011 and 2012, perhaps due to less variation in oiling conditions and incomplete vegetation recovery across our oiled study area.

Reviewing the wave model output and discussion from Silliman *et al*. (2012) [28], as well as fetch and wind speed and direction data for the study period [66–67], there was likely little difference in wave exposure among the heavily oiled plots and reference plots, reinforcing the idea that differential shoreline retreat was related to oiling and vegetation impacts. Furthermore, review of data from Penland *et al*. (2001) [68] and Couvillion *et al*. (2010) [69] indicate that, prior to the oil spill, there were no substantial differences in rates of shoreline retreat between the heavily oiled plots and the reference plots for the 78-year period from 1932 to 2010 (1.2 m yr^-1^ vs. 1.3 m yr^-1^, respectively). These data further reinforce the notion that accelerated shoreline retreat in 2011 and 2012 was related to oiling, rather than differences in plot location, aspect, wave energy, or other factors.

In addition to oiling effects on erosion, we found that the manual treatments did not result in further accelerated shoreline retreat relative to no treatment, a major concern during oil removal actions in marshes. In addition, the greater vegetation recovery observed through 2012 for manual treatment may lessen future shoreline retreat linked to the oil spill. *S*. *alterniflora* recovery in particular may be important in this regard, since it is the typical dominant salt marsh species in the region. Improved *S*. *alterniflora* recovery would also be important if *S*. *alterniflora* provides greater shoreline stabilization compared to other species, such as *P*. *vaginatum* and *D*. *spicata*, which are shorter in stature and more shallowly or weakly rooted [51,70].

Overall, if the oil spill resulted in accelerated shoreline retreat over a wider scale, even for a few years, this would equate to permanent or longer-term marsh habitat loss and comparable losses and impacts for associated resources, beyond those caused by direct oiling of marsh habitat and biota. These permanent or longer-term losses would apply to marsh vegetation, soils, marsh periwinkles, fiddler crabs, and other species dependent on salt marshes (as well as other ecosystem components and services). In the present study, our findings are based on oiled marsh areas present (not eroded) at the time of sampling in 2011 and 2012. Losses of vegetation, marsh periwinkles, and fiddler crabs to erosion have not been estimated.

### Post-Treatment Planting

In 2012, roughly one year after planting, *S*. *alterniflora* cover was 69% (±10 SE) in the planted plots. Qualitative comparison with the main test plots for the same time period indicated that planting resulted in much greater vegetation cover (faster vegetation recovery) compared to any of the treatments without planting (Figs 7 and 8, S1 Table). For comparison, total vegetation cover was 23% (±8 SE) and *S*. *alterniflora* cover was 9% (±5 SE) in the main mechanical treatment plots in 2012, where vegetation was not planted (Figs 7 and 8). In a related ongoing *Deepwater Horizon* study, we reported vegetation impacts extending through 2013 in areas without planting (more than three years following heavy oiling) [52]. In contrast, sites planted with *S*. *alterniflora* had similar vegetation cover, 88% (±3 SE), compared to reference conditions at two years post-planting [52].

**Fig 15:**
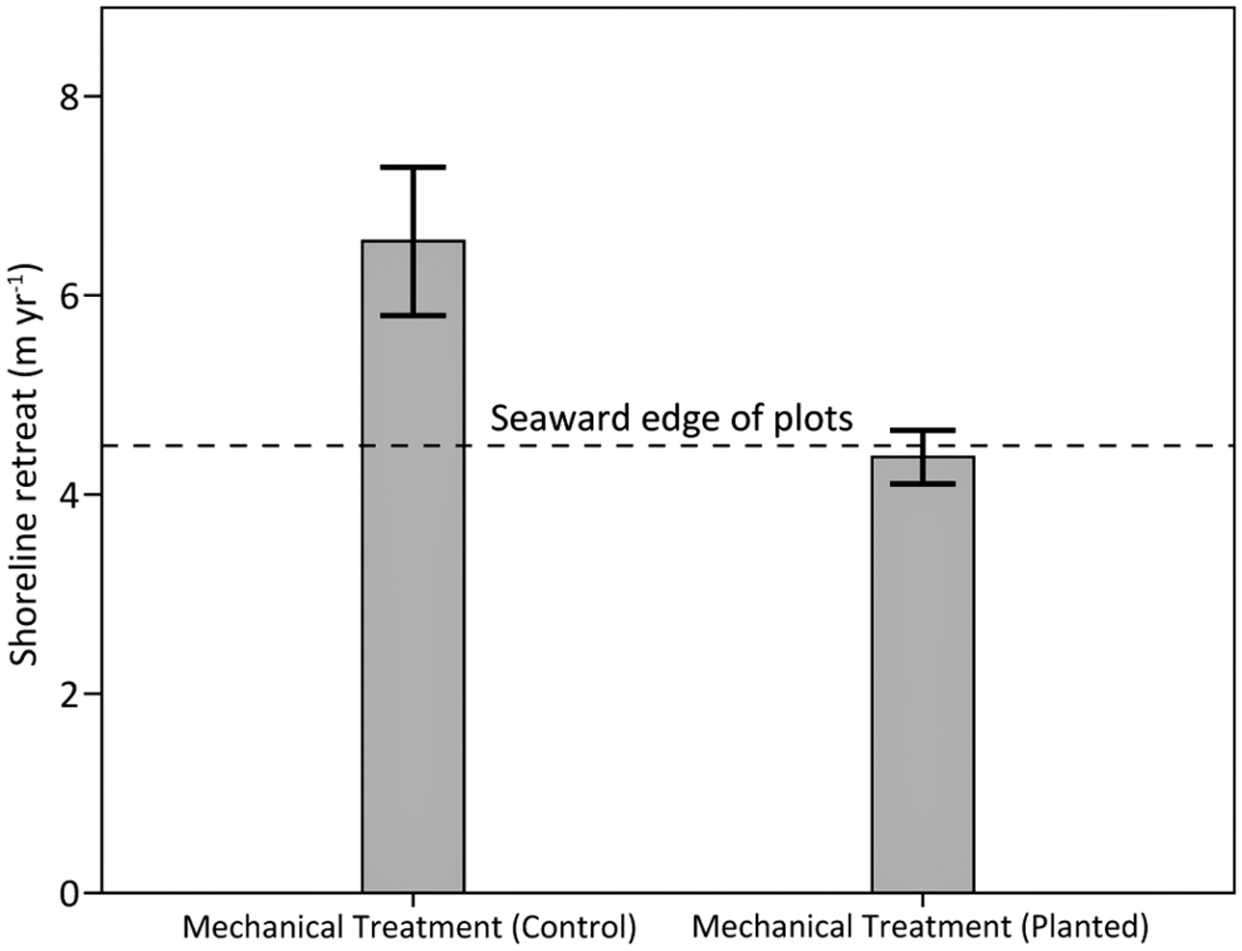
Marsh shoreline retreat among planted and control (unplanted) plots in mechanically treated areas in 2012. Differences were observed for planted versus controls (p = 0.03). Data are means ± 1 SE. The dashed line represents the position of the seaward edge of the plots, which was also the seaward edge of vegetation in the planted plots. N = 5 for both planted and control plots.

The rate of shoreline retreat was 1.5 times greater for the unplanted control plots compared to the planted plots (Fig 15). In addition, shoreline retreat progressed to the seaward edge of the planted plots and halted where the vegetation began, but continued for an additional ~2 m into the interior of the unplanted control plots. These results indicate a clear positive influence of *S*. *alterniflora* in slowing erosion in areas with heavy oiling and mechanical treatment. The influence of marsh vegetation in slowing erosion has been reviewed elsewhere [71–72]. *S*. *alterniflora* may slow erosion through a variety of mechanisms, including baffling wave energy, trapping sediments, and strengthening or binding marsh soils by roots and rhizomes. Over time, the direct and indirect contribution of *S*. *alterniflora* to the accumulation of organic soil material would also contribute to marsh stability.

Qualitative comparison also indicated that shoreline retreat was greater in the mechanical treatment areas than in the manual treatment and no treatment plots, even with planting, and was 4–5 times greater than in the reference plots (Figs 14 and 15). For instance, shoreline retreat in the areas with mechanical treatment and planting was 4.4 m yr^-1^ (±0.3 SE) compared to 3.1 m yr^-1^ (±0.6 SE) in the no treatment plots in 2012. These results show that not only did oiling accelerate shoreline retreat, but that mechanical treatment likely accelerated shoreline retreat even further, though subsequent vegetation planting lessened this effect. The more aggressive nature of the mechanical treatments, including mechanized raking and scraping, appeared to lower the marsh surface and remove or weaken the remaining root mat comprising the upper marsh platform, making these areas more susceptible to erosion and shoreline loss. Based on our field observations and feedback from monitors engaged in marsh treatment operations, we do not necessarily think that mechanical treatment accelerated shoreline retreat everywhere it was applied (see [2]); however, this does appear to be the case in our study area, where mechanical treatment has been most closely evaluated. Also, operational treatments across the wider area of response were not exclusively mechanical; manual treatments alone were also conducted at operational scale in many shoreline treatment areas under the emergency response.

Given the success of planting and its positive influence on improving vegetation recovery and reducing shoreline retreat, we recommend that planting be considered under similar conditions, particularly where vegetation impacts are substantial, vegetation recovery is slow, background erosion rates are high, or intensive treatments are used. Planting in such cases could be conducted as part of shoreline treatment operations under the emergency response or as emergency restoration under the Natural Resource Damage Assessment (NRDA) process. Planting following heavy oiling and intensive treatment may be especially important in places such as coastal Louisiana, where background marsh erosion rates are high and coastal marsh loss is a major concern [23–24]. When planting is evaluated for use, residual oiling levels, soil toxicity, and plant species tolerances should be considered as this could affect planting success [73–74]. In this study, though residual oiling levels were relatively high, the sites were within the tolerance limits of *S*. *alterniflora* reported for South Louisiana crude oil in marsh soils [2,73]. Finally, the influence of planting on a variety of other marsh characteristics and resources, including marsh fauna, should be examined in more detail.

## Conclusions

**Fig 16:**
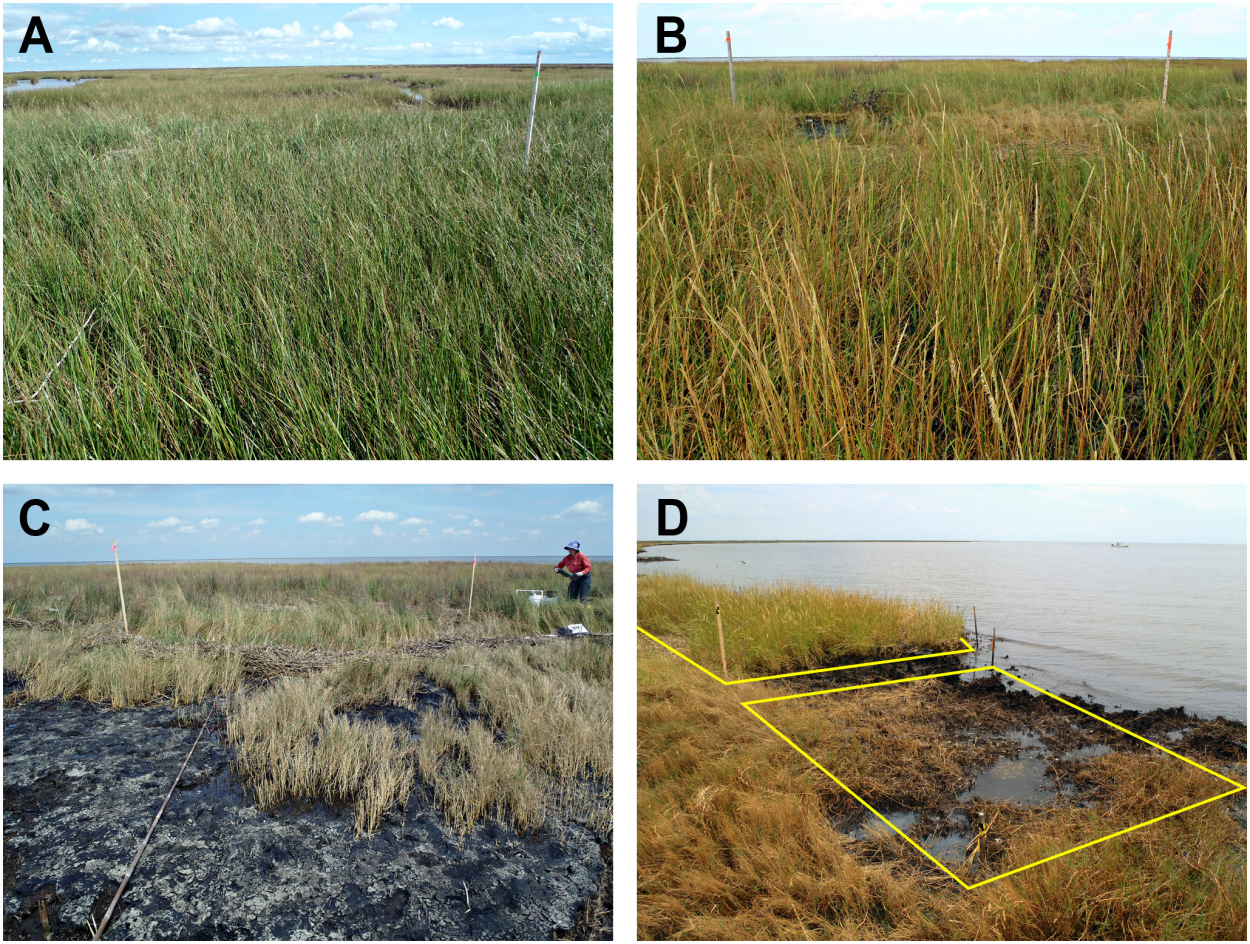
Visual summary of marsh conditions in 2012, more than two years following heavy oiling. (A) Reference plot, (B) heavily oiled plot with manual treatment, (C) heavily oiled plot with no treatment, (D) heavily oiled plots with mechanical treatment, with planting (upper left) and without planting (middle).

Negative effects of persistent heavy oiling on marsh vegetation, intertidal invertebrates, and erosion were ongoing as of 2012 (Fig 16 provides a visual summary across oiling/treatment classes). In areas that were not treated, oiling conditions and negative effects for most ecological parameters did not considerably improve over two years. Both manual and mechanical treatment were effective at improving oiling conditions and vegetation characteristics, beginning the recovery process, though recovery was not complete by two years. Mechanical treatment had additional negative effects of mixing oil into the marsh soils and further accelerating erosion. Manual treatment appeared to strike the right balance between improving oiling and habitat conditions while not causing additional detrimental effects, consistent with prior guidance from case histories [5,11,13]. However, even with these improvements, marsh periwinkles were reduced and showed minimal signs of recovery through two years. These results indicate that definitions of impact and recovery should not be limited to marsh vegetation, and that some ecosystem components may lag the recovery of vegetation structure. Planting following treatment accelerated vegetation recovery and reduced marsh erosion in areas with mechanical treatment. Additional benefits of post-treatment planting on other marsh characteristics warrant further study. Faced with comparable persistent heavy marsh oiling in the future, we would recommend manual treatment followed by planting. We emphasize, however, that the intensive treatments methods examined here (raking, cutting, etc.) would not be appropriate for the majority of oiled marshes, particularly those with lighter or non-persistent oiling, and could result in further marsh damage and delayed recovery, especially if not carefully monitored. Natural recovery (no treatment) would be an appropriate approach for many oiled marshes, especially those with lesser oiling. Oiled controls (no treatment “set-asides”) are essential for judging marsh treatment effectiveness and ecological effects; we recommend their use when testing or applying intensive or alternative treatment methods.

## Supporting Information

S1 Fig Pre-treatment oiled vegetation mat cover in 2010. Differences among oiling/treatment classes were not observed (p = 0.28). Data are means ± 1 standard error (SE). N = 9 for the heavily oiled plots with no treatment; n = 5 for all other oiling/treatment classes.
(TIF)

S2 Fig Pre-treatment surface oil cover in 2010. Differences among oiling/treatment classes were not observed (p = 0.86). Data are means ± 1 standard error (SE). N = 9 for the heavily oiled plots with no treatment; n = 5 for all other oiling/treatment classes.
(TIF)

S1 Table Means ± SE for all parameters by year and oiling/treatment class. (XLSX)
