# Supplementary figures and images for: Heavily Oiled Salt Marsh following the Deepwater Horizon Oil Spill, Ecological Comparisons of Shoreline Cleanup Treatments and Recovery
Source: PLoS One. 2015 Jul 22;10(7):e0132324. doi: 10.1371/journal.pone.0132324 (PMC4511762; doi:10.1371/journal.pone.0132324)

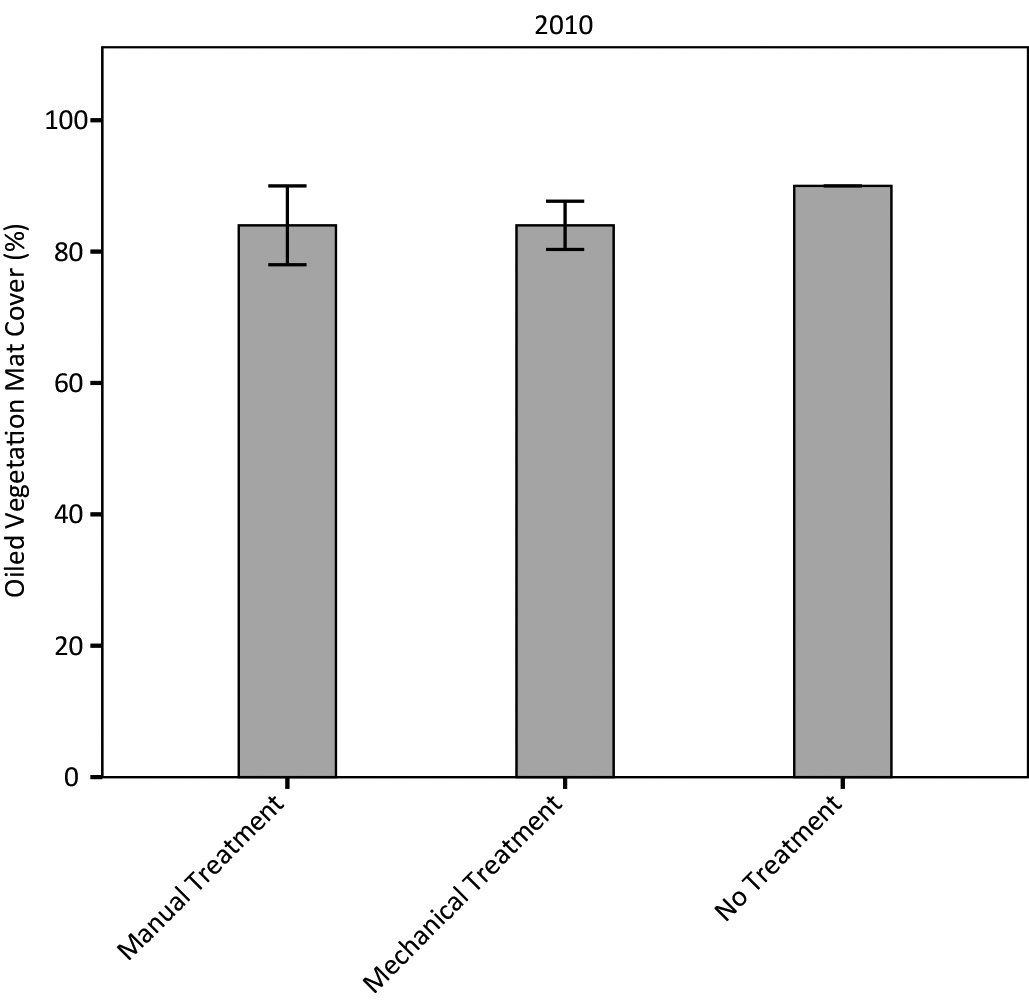

Supplement: S1 Fig — Differences among oiling/treatment classes were not observed (p = 0.28). Data are means ± 1 standard error (SE). N = 9 for the heavily oiled plots with no treatment; n = 5 for all other oiling/treatment classes. (TIF) [file pone.0132324.s001.tif]

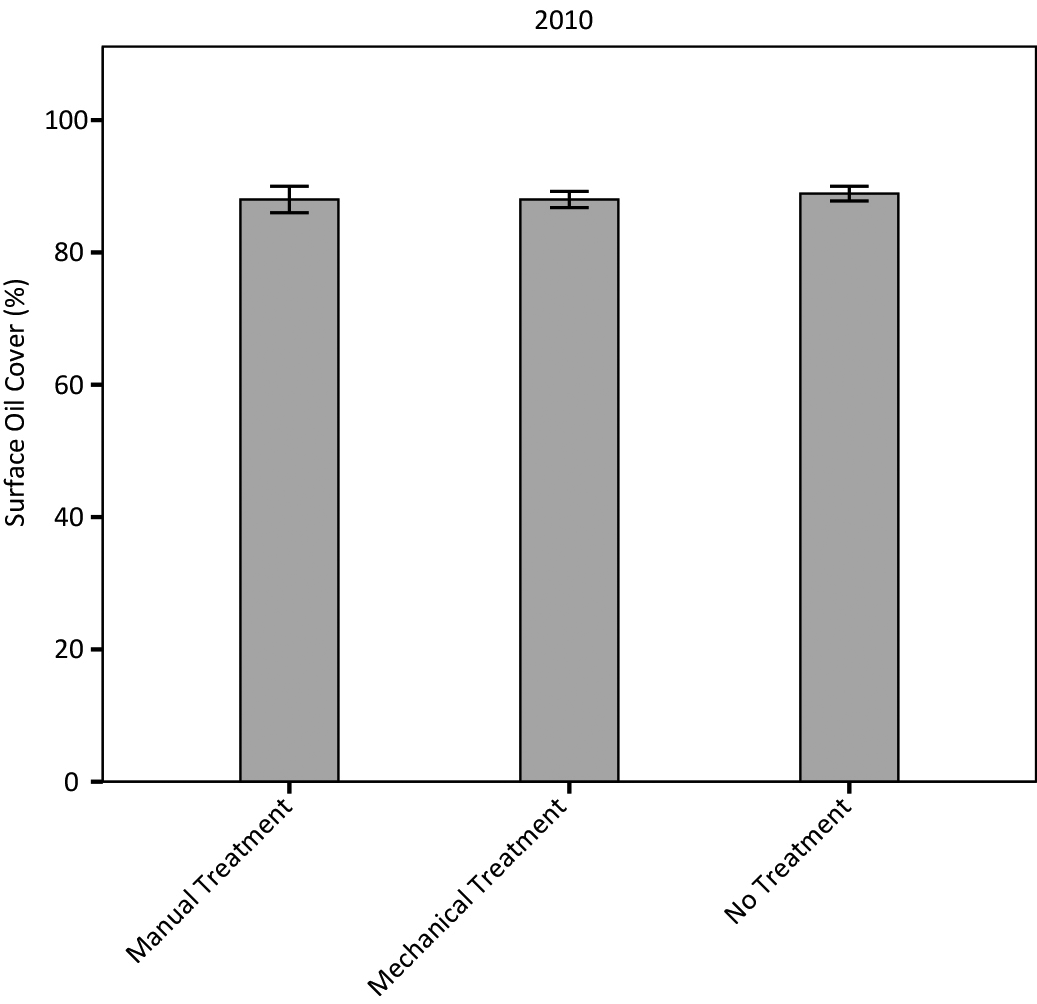

Supplement: S2 Fig — Differences among oiling/treatment classes were not observed (p = 0.86). Data are means ± 1 standard error (SE). N = 9 for the heavily oiled plots with no treatment; n = 5 for all other oiling/treatment classes. (TIF) [file pone.0132324.s002.tif]
